# Placebo effects of repetitive transcranial magnetic stimulation on negative symptoms and cognition in patients with schizophrenia spectrum disorders: a systematic review and meta-analysis

**DOI:** 10.3389/fpsyt.2024.1377257

**Published:** 2024-05-28

**Authors:** Mingqi Wang, Shensen Lu, Lu Hao, Yifei Xia, Zhenchun Shi, Lei Su

**Affiliations:** ^1^ Department of Rehabilitation Medicine, Shandong Mental Health Center, Shandong University, Jinan, China; ^2^ Department of Rehabilitation Medicine, The Second Affiliated Hospital, Zhejiang University School of Medicine, Hangzhou, China

**Keywords:** schizophrenia spectrum disorders, repetitive transcranial magnetic stimulation, placebo effects, negative symptoms, cognition, randomized controlled trial

## Abstract

**Background:**

Negative symptoms and cognitive impairments are highly frequent in schizophrenia spectrum disorders (SSD), associated with adverse functional outcomes and quality of life. Repetitive transcranial magnetic stimulation (rTMS) has been considered a promising therapeutic option in SSD. However, placebo effects of rTMS on these symptoms remained unclear.

**Objective:**

To investigate placebo effects of rTMS on alleviating negative symptoms and cognitive impairment in patients with SSD and to explore potential moderators.

**Methods:**

We systematically searched five electronic databases up to 15 July 2023. Randomized, double-blind, sham-controlled trials investigating effects of rTMS on negative symptoms or cognition in patients with SSD were included. The pooled placebo effect sizes, represented by Hedges’ g, were estimated using the random-effects model. Potential moderators were explored through subgroup analysis and meta-regression.

**Results:**

Forty-four randomized controlled trials with 961 patients (mean age 37.53 years; 28.1% female) in the sham group were included. Significant low-to-moderate pooled placebo effect sizes were observed for negative symptoms (g=0.44, *p<*0.001), memory (g=0.31, *p=*0.010), executive function (g=0.35, *p<*0.001), working memory (g=0.26, *p=*0.004), and processing speed (g=0.36, *p=*0.004). Subgroup analysis indicated that placebo effects were affected by sham stimulation methods, rTMS targeting approaches, and stimulation frequency.

**Conclusions:**

Placebo effects of rTMS on negative symptoms and cognition in patients with SSD are significant in a small-to-moderate magnitude, which might be mediated by rTMS parameters. Our findings will provide new insights for practitioners to further optimize and establish standardized rTMS protocols for future RCTs tackling cardinal symptoms in SSD.

**Systematic Review Registration:**

https://www.crd.york.ac.uk/prospero/, identifier CRD42023390138.

## 1 Introduction

Schizophrenia spectrum disorders (SSD) are devastating neuropsychiatric illnesses with a prevalence of around 3% (1). Cardinal symptoms of SSD include positive symptoms such as delusions and hallucinations, negative symptoms, and cognitive deficits (2). Patients with negative symptoms experience prolonged social withdrawal and decreased interests, which lead to adverse quality of life (3). In addition, cognitive deficits are strongly associated with poor functional outcomes, such as poor ability to live independently or interact with other people (4). It has been reported that 20% to 30% of patients with SSD after pharmacotherapy have residual positive symptoms (5, 6). However, the percentage of patients who still experience residual negative symptoms after long-term antipsychotic treatment is as high as 50% (7, 8). Moreover, a prospective study with a 15-year follow-up showed that cognitive deficits in people with first-episode SSD lasted for more than 15 years (9). These reports reflect necessity of advancing therapy for negative symptoms and cognitive deficits in patients with SSD.

Repetitive transcranial magnetic stimulation (rTMS), a popular non-invasive brain stimulation technique with few side effects such as mild headache (10), is considered promising intervention for ameliorating symptoms in patients with SSD (11). A meta-analysis by Hyde et al. (11) analyzed 59 RCTs and found that active rTMS was significantly more effective than sham rTMS in improving total symptoms, auditory hallucinations, and negative symptoms in individuals with schizophrenia or schizoaffective disorder. rTMS delivers magnetic field pulses to a focused region of the scalp to induce changes in neural activity of the underlying brain areas (12). Because of its promising effectiveness on modulating targeted neural activity and further reducing symptoms (12), increasing research attention has been attracted to examining effects of rTMS on negative symptoms and cognitive deficits in patients with SSD (13, 14). A meta-analysis report published in 2018 (15) has showed that rTMS administrated to the dorsolateral prefrontal cortex induces an overall moderate effect on lessening severity of negative symptoms in patients with SSD. In addition, previous studies have indicated that high-frequency rTMS targeting the dorsolateral prefrontal cortex effectively improves working memory and social cognition in patients with SSD (14, 16). It is noteworthy that even though beneficial effects of rTMS on negative and cognitive symptoms have been indicated, accumulated studies (17, 18) have pointed out a possibility of placebo effects of rTMS.

Placebo effects refer to positive responses in patient’s symptoms or clinical outcomes after receiving a sham treatment (e.g. inert pills, sham neuromodulation or saline injections) as a control in randomized sham-controlled trials (RCT) (19). In research contexts, placebo effects are typically measured as the change in outcome measure compared to baseline after administration of a sham treatment in the sham group (17, 19). Accumulating number of clinical studies and meta-analyses have reported placebo effects existing in the treatment of psychiatric disorders such as depression (17, 20), resistant obsessive-compulsive disorder (21), primary insomnia (22), and auditory hallucinations (23). It has been shown that placebo effects caused by rTMS are attributable to a range of sham stimulation methods (e.g. sham coil or active coil positioned at 45°, 90° or 180° from the skull) used in RCTs, which are associated with patient’s expectations of symptom improvement and memories of former treatment experiences (24). Moreover, rTMS targeting approaches (e.g. 10–20 EEG location system and MRI-neuronavigation system), stimulation frequency, patient characteristics (e.g. age, gender and treatment duration), and study design (e.g. number of research centers) are also possible moderators modifying placebo effects (25). Investigating factors that mediate placebo effects will be beneficial in establishing precise strategies for controlling components that influence placebo effects, optimizing placebo procedures of rTMS, and facilitating the development of novel rTMS protocols in SSD.

Currently, although a growing body of research shows positive effects of rTMS on negative symptoms and cognitive impairments in patients with SSD, no systematic review and meta-analysis has examined the important issue of placebo effects of rTMS. To our best knowledge, only one meta-analysis of 21 RCTs by Dollfus et al. (23) in 2016 has investigated placebo effects of rTMS on auditory hallucinations in patients with SSD, which found that placebo effects on hallucinations were small but evident (Hedges’ g=0.29) and related to sham stimulation methods as well as study design (i.e. parallel-group or crossover). However, it remains unclear how large placebo effects of rTMS are on the remaining cardinal symptoms represented by negative symptoms and cognitive deficits, and which moderators influence the effects.

To sum up, the purpose of this study was to conduct a systematic review and meta-analysis to examine placebo effects of rTMS on negative symptoms and cognitive impairment in patients with SSD. The secondary purpose was to identify possible moderators of the aforementioned placebo effects of rTMS. We believe that elucidating placebo effects of rTMS and associated moderators would help researchers to develop more efficacious rTMS protocols with less placebo effects in treatment of negative and cognitive symptoms. Meanwhile, our results would guide clinicians to improve placebo procedures of rTMS and to apply more effective treatment parameters for core symptoms of SSD in clinical practice.

## 2 Methods

This report was conducted and reported in accordance with the Preferred Reporting Items for Systematic Reviews and Meta-Analyses statement (26). The protocol for this study was registered in PROSPERO with the ID CRD42023390138 (https://www.crd.york.ac.uk/prospero/).

### 2.1 Search strategy and eligibility criteria

Two authors of this study searched papers collected by the Cochrane Library, Medline, Web of Science, CINAHL, and EMBASE until July 15th, 2023 according to a set of key words (**Supplementary Table S1**). We also manually screened references of included publications for identifying eligible articles.

This study included randomized, double-blind, sham-controlled, parallel-group, and cross-over trials using sham rTMS as a control compared with real rTMS. Participants had to be diagnosed with SSD according to the Diagnostic and Statistical Manual of Mental Disorders (27), the International Classification of Diseases (28) or the Mini International Neuropsychiatric Interview (29). Study outcomes are cognition and/or negative symptoms. Publications were ineligible if they were not written in English, or did not have full-text or raw data available for effect size calculation after we contacted the corresponding authors of the publications by e-mail. Titles and abstracts of publications were independently screened to identify eligibility for full-text evaluation after duplicate publications were removed. Any discrepancies during this process were discussed. The persistent discrepancies were adjudicated by the corresponding author.

### 2.2 Data extraction

We used a standardized spreadsheet to collect relevant data, including first author, publication year, study design, demographic characteristics, sample size of the sham group, rTMS parameters of the sham group, and outcome measures. We defined placebo effects as the mean difference of outcome measures from baseline to the end of treatment in the sham group (30). Therefore, results of outcome measures such as mean and standard deviations at baseline and after intervention in the sham group were collected in detail. Detailed information is shown in **Table 1**. During the process of data extraction, if a study assumed multiple instruments for the evaluation of a single outcome, the data were collected from the primary outcome as specified by the study authors. In the absence of a clearly defined primary outcome, the data were collected from the instrument that was most commonly investigated. Detailed information about outcome measures employed in each study can be found in **Table 1**. When an outcome was assessed multiple times, data from the end of treatment were selected for analysis, as in previous studies (73, 74). Moreover, if data were unclear and could not be extracted from graphs, we contacted the corresponding author by e-mail to obtain the data. If there was no response, studies were excluded.

**Table 1:** Characteristics of included RCTs.

Author	sample size in sham group (female)	Mean age (Illness duration)	Coil type (sham condition)	Stimulation targets	Methods of target localization	Stimulation parameters	Treatment duration	Evaluation time points	Outcome measures (domains)
Barr et al. (2011) (31)	12 (5)	47.21 (NA)	A 70-mm figure-of-eight coil (coil flipped 90°)	Bilateral DLPFC	MRI-neuro navigation	20 Hz, 90% RMT, 750 pulses (per hemisphere)	1 session in 1 day	baseline (T0), end point of treatment (T1)	n-back (working memory)
Barr et al. (2012) (32)	12 (2)	47.92 (27.30)	A 70-mm figure-of-eight coil (coil flipped 90°)	Bilateral DLPFC	MRI-neuro navigation	20Hz, 90% RMT, 1500 pulses	20 sessions, 4 weeks (5 days/week)	baseline (T0), 1 (T1), 2 (T2), 3 (T3), end point of treatment (T4), and 2 weeks (T5) follow-up	SANS (negative symptoms)
Barr et al. (2013) (16)	14 (3)	49.00 (24.50)	A 70-mm figure-of-eight coil (coil flipped 90°)	Bilateral DLPFC	MRI-neuro navigation	20Hz, 90% RMT, 1500 pulses	20 sessions, 4 weeks (5 days/week)	baseline (T0), end point of treatment (T1)	n-back (working memory)
Basavaraju et al. (2021) (33)	30 (0)	NA(NA)	A figure-of-eight coil (sham coil)	Cerebellar vermis	MRI-neuro navigation	iTBS (50Hz), 100% MT, 600 pulses	10 sessions, 5 days (2 sessions/day)	baseline (T0), end point of treatment (T1), and 37 days (T2) follow-up	SANS (negative symptoms)
Bation et al. (2021) (34)	10 (1)	41.60 (17.11)	A figure-of-eight coil (sham coil)	Left DLPFC	Anatomical location	iTBS (50Hz), 80% RMT, 990 pulses	20 sessions, 2 weeks (2 sessions/weekday)	baseline (T0), end point of treatment (T1), and 1 (T2), 3 (T3), and 6 (T4) months follow-up	SANS (negative symptoms)
Bodén et al. (2021) (35)	28 (12)	31.30 (NA)	A combined active/placebo coil with two identical sides (sham coil)	DMPFC	MRI-neuro navigation	iTBS (50Hz), 90% RMT, 1200 pulses	20 sessions, 2 weeks (2 sessions/weekday)	baseline (T0), end point of treatment (T1), and 4 weeks follow-up (T2)	CAINS (negative symptoms)
Chauhan et al. (2020) (36)	17 (9)	39.35 (13.00)	A figure-of-eight coil (sham coil)	Cerebellar vermis	10–20 EEG system	iTBS (50Hz), 80% RMT, 1200 pulses	10 sessions, 5 days (2 sessions/day)	baseline (T0), end point of treatment (T1), and 2 weeks follow-up (T2)	PANSS (negative symptoms)
Du et al. (2022) (37)	22 (11)	45.10 (19.90)	A figure-of-eight coil (coil was flipped 180°)	Left DLPFC	10–20 EEG system	10Hz, 110% MT, 1500 pulses	20sessions (1 session/day), 4 weeks (5 days/week)	baseline (T0), end point of treatment (T1), and 4-week follow-up (T2)	PRM-CANTAB (memory); SANS (negative symptoms)
Fitzgerald et al. (2008) (38)	8 (2)	33.20 (6.90)	A 70-mm figure-of-eight coil (coil was flipped 90°)	Bilateral DLPFC	Anatomical location	10 Hz, 110% RMT, 1000 pulses	15 sessions, 3 weeks (5 days/week)	baseline (T0), end point of treatment (T1)	SANS (negative symptoms)
Francis et al. (2019) (39)	10 (2)	22.30 (3.10)	A modified figure-of-eight coil (sham coil)	Bilateral DLPFC	Anatomical location	20Hz, 110% RMT, 600 pulses (per hemisphere)	10 sessions, 2 weeks (5 days/week)	baseline (T0), endpoint of treatment (T1), 2 weeks follow-up (T2)	BACS (memory, executive function, processing speed)
Garg et al. (2016) (40)	20 (4)	30.75 (6.05)	A double-angled cone coil (coil was flipped 45°)	Cerebellar vermis	Anatomical location	5, 6, and 7 Hz, 100% RMT, 600 pulses	10 sessions, 2 weeks (5 days/week)	baseline (T0), end point of treatment (T1), and 2 weeks follow-up (T2)	PANSS (negative symptoms)
Guan et al. (2020) (41)	28 (0)	56.00 (34.50)	A Mag-Venture CoolB65 Active/placebo coil (placebo coil)	Left DLPFC	MRI-neuro navigation	20 Hz, 110% MT, 1600 pulses	40 sessions, 8 weeks (5 days/week)	baseline (T0), week 2 (T1), week 4 (T2), week 6 (T3), and end point of treatment (T4)	RBANS (memory, attention, visuospatial function, language); PANSS (negative symptoms)
Güleken et al. (2020) (42)	10 (4)	34.40 (11.09)	A 70-mm figure-of-eight coil (coil was flipped 90°)	Bilateral DLPFC	NA	20 Hz, 90% RMT, 1000 pulses	20 sessions, 4 weeks (5 days/week)	baseline (T0), end point of treatment (T1)	SCWT (executive function); DST (working memory)
Guse et al. (2013) (43)	12 (3)	36.00 (NA)	A standard figure-of-eight coil (coil was flipped 45°)	Left DLPFC	10–20-EEG system	10 Hz, 110% RMT, 1000 pulses	15 sessions, 3 weeks (5 days/week)	baseline (T0), end point of treatment (T1)	n-back (working memory); TMT-A (processing speed); TMT-B, WCST (executive function); TAP (attention)
Hasan et al. (2016) (44)	79 (22)	35.50 (NA)	A cooled MCF-B65 figure-of-eight coil (coil was tilted 45°)	Left DLPFC	10–20-EEG system	10Hz, 110% RMT, 1000 pulses	15 sessions, 3 weeks (5 days/week)	baseline (T0), end point of treatment (T1), 24-day (T2), and 81-day (T3) follow-up	Verbal Learning and Memory Test (memory); TMT-A (processing speed); WCST, Regensburg word fluency test (executive function)
Holi et al. (2004) (45)	11 (0)	34.80 (12.90)	A 70-mm figure eight-shaped coil (coil was flipped 90°)	Left DLPFC	Anatomical location	10Hz, 100% MT, 1000 pulses	10 sessions, 2 weeks (5 days/week)	baseline (T0), end point of treatment (T1)	PANSS (negative symptoms)
Huang et al. (2016) (46)	18 (0)	39.39 (28.67)	A 70-mm figure eight-shaped coil (sham coil)	Left DLPFC	Anatomical location	10 Hz, 110% MT, 2000 pulses	21 sessions, 3 weeks (7 days/week)	baseline (T0), end point of treatment (T1)	WCST (executive function); PANSS (negative symptoms)
Jin et al. (2023) (12)	32 (13)	47.47 (8.40)	A 70-mm air-cooled butterfly coil (sham coil)	Left DLPFC	MRI-neuro navigation	iTBS (50Hz), 120% RMT, 1800 pulses	60 sessions (3 sessions/day), 4 weeks (5 days/week)	baseline (T0), week 2 (T1), and end point of treatment (T2)	PANSS (negative symptoms)
Klein et al. (1999) (47)	17 (11)	29.50 (7.90)	A 9-cm external diameter circular coil (coil was flipped 90°)	Right prefrontal area	Anatomical location	1Hz, 110% MT, 870 pulses	10 sessions, 2 weeks (5 days/week)	baseline (T0), end point of treatment (T1)	PANSS (negative symptoms)
Kumar et al. (2020) (48)	50 (22)	30.80 (8.46)	A figure-of-8 Double Rapid2 Air Cooled Coil (sham coil)	Left DLPFC	Anatomical location	20 Hz, 100% MT, 2000 pulses	20 sessions, 4 weeks (5 days/week)	baseline (T0), end point of treatment (T1), and 1 (T2), 2 (T3), 3 (T4), and 4 (T5) months follow-up	SANS (negative symptoms)
Lange et al. (2015) (49)	16 (4)	32.30 (9.92)	A 75-mm figure-of-eight coil (coil was flipped 90°)	Bilateral DLPFC	10–20 EEG system	10Hz, 90% MT, 2000 pulses (per hemisphere)	30 sessions, 3 weeks (5 days/week)	baseline (T0), end point of treatment (T1), 4 weeks (T2), and 3 months (T3) follow-up	TMT-A (processing speed); TMT-B, WCST (executive function); Digit symbol substitution test (processing speed); SANS (negative symptoms)
Li et al. (2016) (50)	22 (11)	49.90 (19.00)	NA (sham coil)	Left DLPFC	NA	10Hz, 110% RMT, 1500 pulses	20 sessions, 4 weeks (5 days/week)	baseline (T0), end point of treatment (T1), and 4 weeks follow-up (T2)	SANS (negative symptoms)
McIntosh et al. (2004) (51)	16 (9)	35.90 (NA)	A 70-mm figure-of-eight coil (coil was flipped 45°)	Left temporo-parietal cortex	10–20 EEG system	1Hz, 80%MT	4 sessions, 4 days	baseline (T0), end point of treatment (T1)	Verbal learning test (memory); PANSS (negative symptoms)
Mittrach et al. (2010) (52)	14 (3)	34.40 (5.60)	A 100-mm figure-eight coil (sham coil)	Left DLPFC	Anatomical location	10Hz, 110% MT, 1000 pulses	10 sessions, 2 weeks (5 days/week)	baseline (T0), end point of treatment (T1)	D2-attention task (attention); WCST (executive function); TMT-A (processing speed); TMT-B (executive function)
Mogg et al. (2007) (53)	9 (0)	33.60 (9.00)	A figure-of-eight coil (sham coil)	Left DLPFC	Anatomical location	10Hz, 110% RMT, 2000 pulses	10 sessions, 2 weeks (5 days/week)	baseline (T0), end point of treatment (T1), and 2 weeks follow-up (T3)	Controlled oral word association test (executive function); Verbal learning memory test (memory); PANSS (negative symptoms)
Pan et al. (2021) (54)	19 (7)	57.37 (29.37)	A figure-of-eight coil (sham coil)	Left DLPFC	10–20 EEG system	10Hz, 110% RMT, 1600 pulses	20 sessions, 4 weeks (5 days/week)	baseline (T0), end point of treatment (T1)	PANSS (negative symptoms)
Prikryl et al. (2007) (55)	11 (0)	36.46 (8.18)	NA (coil was flipped 90°)	Left DLPFC	NA	10Hz, 110% RMT, 1500pulses	15 sessions, 3weeks(5days/week)	baseline (T0), end point of treatment (T1)	SANS (negative symptoms)
Prikryl et al. (2012) (56)	11 (0)	34.55 (4.18)	A figure-of-eight coil (sham coil)	Left DLPFC	10–20 EEG system	10Hz, 110% MT, 1500 pulses	15 sessions, 3 weeks (5 days/week)	baseline (T0), end point of treatment (T1)	Verbal Fluency Task (working memory); PANSS (negative symptoms)
Prikryl et al. (2013) (57)	17 (0)	33.94 (5.89)	A figure-of-eight coil (sham coil)	Left DLPFC	Anatomical location	10Hz, 110% MT, 2000 pulses	15 sessions, 3 weeks (5 days/week)	baseline (T0), end point of treatment (T1)	SANS (negative symptoms)
Prikryl et al. (2014) (58)	17 (0)	34.58 (4.13)	A figure-of-eight coil (sham coil)	Left DLPFC	Anatomical location	10Hz, 110% MT, 2000 pulses	15 sessions, 3 weeks (7 days/week)	baseline (T0), end point of treatment (T1)	PANSS (negative symptoms)
Quan et al. (2015) (59)	39 (11)	46.87 (17.97)	A 9-cm circular coil (coil was flipped 90°)	Left DLPFC	Anatomical location	10Hz, 80% MT, 800 pulses	20 sessions, 2 weeks (5days/week), 2weeks interval, and 2 weeks (5 days/week)	baseline (T0), week 2 (T1), end point of treatment (T2)	SANS (negative symptoms)
Rabany et al. (2014) (60)	10 (2)	35.90 (14.00)	An H1 deep-TMS coil (sham coil)	Left DLPFC	Anatomical location	20Hz, 120% MT, 1680 pulses	20 sessions, 4 weeks (5 days/week)	baseline (T0), end point of treatment (T1), 1-month (T2) follow-up	SANS (negative symptoms); Rapid visual information processing (attention); PRM (memory); Stocking of Cambridge (executive function); Special working memory test (working memory)
Saba et al. (2006) (61)	8 (0)	30.60 (8.20)	A figure-of-eight coil (sham coil)	Left temporo-parietal cortex	10–20 EEG system	1HZ, 80% MT, 300 pulses	14 sessions, 2 weeks (5 days/week)	baseline (T0), end point of treatment (T1)	PANSS (negative symptoms)
Singh et al. (2020) (62)	15 (6)	29.80 (9.50)	A 70-mm figure-of-eight air-film coil (sham coil)	Left DLPFC	Anatomical location	20Hz, 100% MT, 2000 pulses	20 sessions, 4 weeks (5 days/week)	baseline (T0), day 5 (T1), and end point of treatment (T2)	SANS (negative symptoms)
Su et al. (2022) (63)	19 (0)	55.60 (34.50)	A 70-mm figure-of-eight coil (sham coil)	Left DLPFC	MRI-neuro navigation	10 Hz, 110% MT, 1200 pulses	20 sessions, 4 weeks (5 days/week)	baseline (T0), end point of treatment (T1)	PANSS (negative symptoms)
Tikka et al. (2017) (64)	10 (0)	25.50 (3.45)	An air-cooled figure-of-eight coil (sham coil)	Right inferior parietal lobule	MRI-neuro navigation	50Hz, 80% RMT, 900 pulses	10 sessions, 2 weeks (5 days/week)	baseline (T0), end point of treatment (T1)	Control task (memory); PANSS (negative symptoms)
Wang et al. (2022) (65)	26 (15)	24.15 (4.62)	A 70-mm air-cooled figure-eight coil (sham coil)	Left DLPFC	MRI-neuro navigation	iTBS (50Hz), 80% RMT, 1800 pulses	42 sessions (3 sessions/day), 14 days	baseline (T0), end point of treatment (T1)	n-back (working memory); SANS (negative symptoms)
Wen et al. (2021) (66)	26 (12)	38.80 (14.40)	A figure-eight-shaped coil (coil was flipped 45°)	Left DLPFC	Anatomical location	10Hz, 110% MT, 1600 pulses	20sessions (1 session/day), 4 weeks (5 days/week)	baseline (T0), end point of treatment (T1)	PANSS (negative symptoms); RBANS (memory, attention, visuospatial function, language); SCWT (executive function)
Wobrock et al. (2015) (67)	81 (25)	34.90 (NA)	A cooled figure-of-eight coil (coil was flipped 45°)	Left DLPFC	10–20 EEG system	10Hz, 110% RMT, 1000 pulses	15 sessions, 3 weeks (5 days/week)	baseline (T0), end point of treatment (T1)	PANSS (negative symptoms)
Wölwer et al. (2014) (68)	14 (3)	34.40 (5.60)	A figure-of-eight coil (sham coil)	Left DLPFC	Anatomical location	10Hz, 110% MT, 1000pulses	10 sessions, 2 weeks (5 days/week)	baseline (T0), end point of treatment (T1)	D2 attention test (attention); TMT-A (processing speed); TMT-B, WCST (executive function); PANSS (negative symptoms)
Xiu et al. (2020) (69)	40 (0)	54.70 (34.10)	A figure-of-eight-coil (sham coil)	Left DLPFC	MRI-neuro navigation	10Hz and 20Hz, 110% MT, 1200 and 1600 pulses	40 sessions, 8 weeks (5 days/week)	baseline (T0), end point of treatment (T1), and 6 months follow-up (T2)	RBANS (memory, attention, visuospatial function, language)
Zhao et al. (2014) (70)	22 (10)	46.70 (NA)	A butterfly coil/MF-125 round coil (coil was flipped 180°)	Left DLPFC	NA	10Hz, 20Hz, and 50Hz, 80% MT, 2400 pulses	20 sessions, 4 weeks (5 days/week)	baseline (T0), end point of treatment (T1)	SANS (negative symptoms)
Zhu et al. (2021) (71)	32 (18)	35.34 (15.84)	A figure-of-eight cool coil (coil was flipped 180° or 90°)	Cerebellar vermis	Anatomical location	iTBS (50Hz), 100% RMT, 600 pulses	10 sessions (1 session/day), 2 weeks (5 days/week)	baseline (T0), end point of treatment (T1), and 2 (T2), 6 (T3), 12 (T4), and 24 weeks follow-up (T5)	PANSS (negative symptoms)
Zhuo et al. (2019) (72)	27 (8)	30.63 (8.11)	A standard butterfly coil (coil was flipped 180°)	Left DLPFC	10–20 EEG system	20Hz, 90% RMT, 2000 pulses	20 sessions, 20 days	baseline (T0), end point of treatment (T1)	SANS (negative symptoms); MCCB (processing speed, attention/vigilance, working memory, verbal learning, visual learning, reasoning/problem solving, social cognition)

BACS, Brief assessment of cognition in schizophrenia; CAINS, Clinical Assessment Interview for Negative Symptoms; DLPFC, Dorsolateral Prefrontal Cortex; DMPFC, Dorsomedial Prefrontal Cortex; DST, Digit Span Test; MCCB, MATRICS Consensus Cognitive Battery; NA, Not Available; PANSS, Positive and Negative Syndrome Scale; PRM-CANTAB, Pattern recognition memory component of the Cambridge Neuropsychological Test Automated Battery; RBANS, Repeatable Battery for the Assessment of Neuropsychological Status; SANS, Scale for the Assessment of Negative Symptoms; SCWT, Stroop Color and Word Test; TAP, Tübinger Aufmerksamkeitsprüfung; TMT-A and -B, Trail Making Test versions A and B; WCST, Wisconsin Card Sorting Test.

To enable direct cross-comparison between studies assessing different cognitive functioning, cognitive outcomes in this study were specifically divided into five sub-domains, including memory, executive function, working memory, attention, and processing speed based on the Measurement and Treatment Research to Improve Cognition in Schizophrenia (11, 75).

### 2.3 Meta analysis

Meta-analyses were performed using the Comprehensive Meta-Analysis (version 3) software. We calculated Hedge’s g, a standardized mean difference, to determine the placebo effect sizes for negative symptoms and cognition. Specifically, it was calculated using the mean and standard deviation of outcome measure at baseline and at the end of intervention in the sham group. Where the above statistics at baseline and at the end of intervention were not reported, we computed the Hedge’s g using the mean difference and its standard deviation of the end of intervention compared to the baseline in the sham group. All calculations of effect sizes were conducted using the Comprehensive Meta-Analysis. The pre- and post-treatment correlations of the sham group for each study were set to be 0.25, which is a modest correlation (30).The magnitude of the Hedges’ g was defined as small (0.2–0.49), medium (0.5–0.79), or large (≥0.8) according to Cohen’s guideline (76).

Study heterogeneity was examined using the I^2^ statistic, which was classified to low (I^2^ ≤ 50%), medium (I^2^: 51–75%), and high (I^2^>75%) levels (77). Publication bias was assessed using Egger’s tests and funnel plots when 10 or more studies were included (78). The quality (i.e., risk of bias) of the included RCTs was assessed using the Cochrane risk-of-bias tool for randomized trials-version 2 (RoB 2) [29]. This tool classified the risk of bias for each study as high, low, or unclear risk. We assumed random-effects model for data analysis, as stimulation parameters and outcome measures varied between studies (77).

In the sensitivity analysis, we excluded studies with a high risk of bias, as defined according to the Cochrane risk-of-bias tool, to test whether the findings remained unchanged (11). We also examined whether effect size estimates would be stable under the assumption of different levels of correlation (r=0, 0.5 and 0.8) between pretest and posttest data. In addition, four subgroup analyses were run to examine potential moderators of placebo effects, including sham stimulation methods, rTMS targeting approaches, stimulation frequency, and efficacy of active rTMS versus sham rTMS. Subgroup analysis was performed when the number of included RCTs for a particular outcome was two or more (77). Finally, we conducted univariate meta-regressions using a random-effect model to examine whether age, proportion of females, illness duration, sample size, treatment duration, randomization ratio (active vs. sham ratio), and publication time were associated with placebo effects. Meta-regressions were performed when the available studies for a particular outcome was no less than 10 (77). *P<*0.05 (two-tailed) was considered the threshold for statistical significance.

## 3 Results

### 3.1 Literature searches

**Figure 1:**
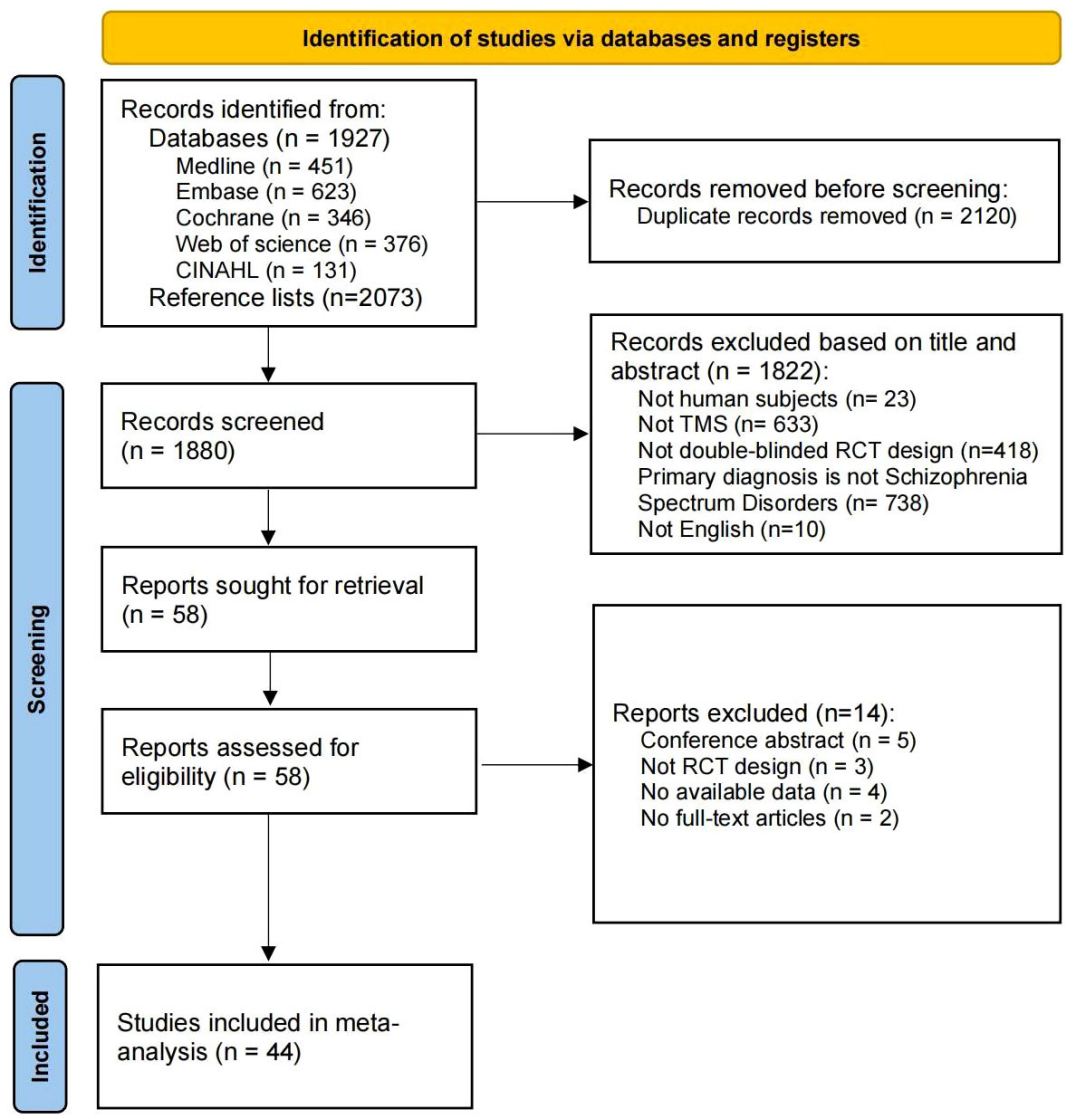
PRISMA flow chart for literature review.

Our systematic search identified 1927 records from five databases and 2073 records from the reference lists of included studies (see **Figure 1**). After excluding 2120 duplicates, 1880 records were examined for title and abstract. Of these, 1822 articles were excluded due to non-TMS, non-double-blind RCT and other reasons. Of the 58 retrieved full-text studies, 44 articles were finally included and analyzed in this review.

### 3.2 Characteristics of included studies

The characteristics of the included studies are listed in **Table 1**. They were reported from 14 countries (China=14, India=6, Germany=5, Czech Republic=4, Canada=3, France=2, Israel=2, UK=2, Australia, Finland, Netherlands, Sweden, Turkey, and USA). The included RCTs recruited a total of 2125 patients (26.26% female) and 961 patients (28.10% female) were allocated to the sham rTMS group, with disease duration ranging from 3.10 to 34.50 years and mean age from 22.30 to 57.37 years. Of these, there were 853 patients with schizophrenia, 9 patients with schizoaffective disorder, 7 patients with schizophreniform disorder and 92 patients who had one of the above three diagnoses but did not be clearly classified by study authors. A total of 34 studies (14, 31, 34, 36–46, 48, 50, 52–56, 58, 59, 61–67, 69–72) assessed severity of illness using the PANSS scale, with a mean total score of 75.06, and two studies (35, 47) used the Brief Psychopathological Rating Scale, with a mean score of 40.30; The remaining studies (16, 32, 33, 49, 51, 57, 60, 68) did not report total scores for the PANSS or the BPRS, which only provide scores for negative symptoms using scales such as the PANSS, the SANS or the CAINS. Additionally, as shown in **Table 1**, sham stimulation methods included sham coils (n=24), coil flipped 45° (n=6), 90° (n=10), or 180° (n=4). rTMS targeting approaches covered anatomical localization (n=18), 10–20 EEG location system (n=11) and MRI-neuronavigation system (n=11). The 8-shaped coil is the most used type, with stimulation frequencies varying between 10Hz, 20 Hz and 50 Hz. The longest duration of rTMS treatment was 60 sessions, while the shortest was a single session. Negative symptoms were reported in 37 studies, memory in 12 studies, executive function in 11 studies, working memory in 9 studies, attention in 7 studies and processing speed in 6 studies.

### 3.3 Methodological quality and publication bias

The quality of the included parallel and cross-over RCTs is shown in **Supplementary Figures S2a**, **S2b**, respectively. Two of the 43 included parallel RCTs exhibited a high risk of bias, 28 studies had a moderate risk of bias and the remaining studies had a low risk of bias. Only one of the included RCTs had a crossover design, which has a low risk of bias.

As there were fewer than 10 RCTs for working memory, attention, and processing speed, we assessed publication bias only for negative symptoms (*p=*0.192, **Supplementary Figure S1a**), memory (*p=*0.802, **Supplementary Figure S1b**), and executive function (*p=*0.694, **Supplementary Figure S1c**) using Egger’s tests and funnel plots. No significant publication bias was found for these outcomes.

### 3.4 Placebo effects of rTMS on negative symptoms

**Figure 2:**
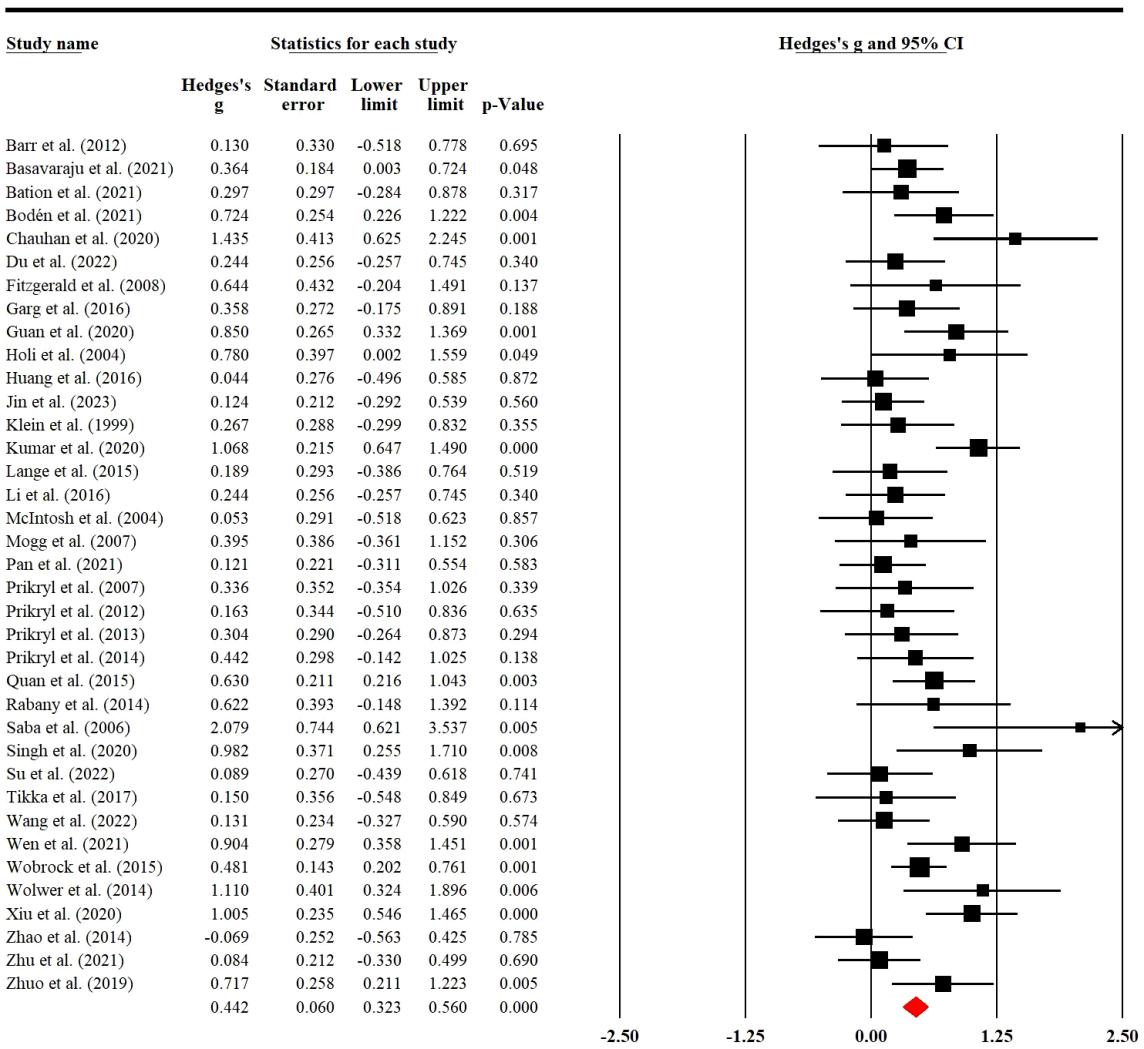
Placebo effects of rTMS on negative symptoms in patients with SSD.

Thirty-seven RCTs involving 810 (228 female) patients were used to examine placebo effects of rTMS on negative symptoms. Meta-analysis indicated that sham rTMS had a significant placebo effect with low heterogeneity on negative symptoms (g=0.44; 95% CI: 0.32 to 0.56; *p<*0.001; I^2^ = 43.12%; **Figure 2**). When we removed two RCTs with a high risk of bias, the result was still stable (g=0.44; 95% CI: 0.32 to 0.57; *p<*0.001; I^2^ = 43.73%; **Supplementary Table S3**).

Subgroup analysis of sham stimulation methods revealed that sham coil, 45°, and 90° position coil (g=0.50, *p<*0.001; g=0.46, *p=*0.002; g=0.35, *p=*0.001; **Supplementary Figure S3a**) significantly affected placebo effects, with the lowest effect size found in the 90° position coil. For rTMS targeting approaches, the placebo effect size of the anatomical localization (g=0.53, *p*<0.001) was higher than that of the 10–20 EEG location system and MRI-neuronavigation system (g=0.47, *p=*0.002; g=0.41, *p=*0.001; **Supplementary Figure S3b**). rTMS at 20-Hz (g=0.82, *p<*0.001; **Supplementary Figure S3c**) had a higher effect than other stimulation frequencies; Moreover, a higher placebo effect size was found in studies with no significant efficacy of active rTMS over sham rTMS (g=0.50, *p<*0.001; **Supplementary Figure S3d**) (see **Table 2**).

**Table 2:** Hedges’ g of subgroup analyses for rTMS trials of placebo effects on negative symptoms and cognition.

Subgroups	Negative symptoms	Memory	Executive function	Working memory	Attention	Processing speed
Sham stimulation methods						
Sham coil	0.50^∗^	0.44	0.21	0.30	0.26	0.08
Angled 45°	0.46^∗^	0.15	0.59^∗^	0.28	0.26	0.30
Angled 90°	0.35^∗^	NA	0.52^∗^	0.32	NA	NA
Angled 180°	0.30	0.43^∗^	NA	NA	NA	NA
Overall	0.43^∗^	0.33^∗^	0.37^∗^	0.30^∗^	0.26	0.26^∗^
rTMS targeting approaches						
MRI-neuronavigation system	0.41^∗^	0.65^∗^	NA	0.24	-0.04	NA
10–20 EEG location system	0.47^∗^	0.15	0.58^∗^	0.26^∗^	0.36	0.44^∗^
Anatomical location	0.53^∗^	0.24	0.22	NA	0.43	0.08
Overall	0.48^∗^	0.31^∗^	0.35^∗^	0.25^∗^	0.24	0.40^∗^
rTMS frequency						
≤5Hz	0.40	NA	NA	NA	NA	NA
10 Hz	0.36^∗^	0.21	0.43^∗^	0.32^∗^	0.35	0.38^∗^
20 Hz	0.82^∗^	0.52	0.17	0.25	0.21	0.18
50 Hz	0.34^∗^	NA	NA	NA	NA	NA
Overall	0.46^∗^	0.24^∗^	0.35^∗^	0.29^∗^	0.24	0.36^∗^
Efficacy of active rTMS over sham rTMS						
YES	0.39^∗^	0.40^∗^	NA	0.27	NA	NA
NO	0.50^∗^	0.27	NA	0.26^∗^	NA	NA
Overall	0.44^∗^	0.36^∗^	NA	0.26^∗^	NA	NA

NA, not available due to the number of included RCTs less than two; ^∗^significant placebo effect, p<0.05; positive Hedges’ g estimate: increased placebo effect; negative Hedges’ g estimate: decreased placebo effect.

Meanwhile, meta-regression indicated that placebo effects of rTMS on negative symptoms was not associated with age, proportion of females, illness duration, treatment days, sample size, randomization ratio, or publication time (see **Supplementary Table S5**).

### 3.5 Placebo effects of rTMS on memory

**Figure 3:**
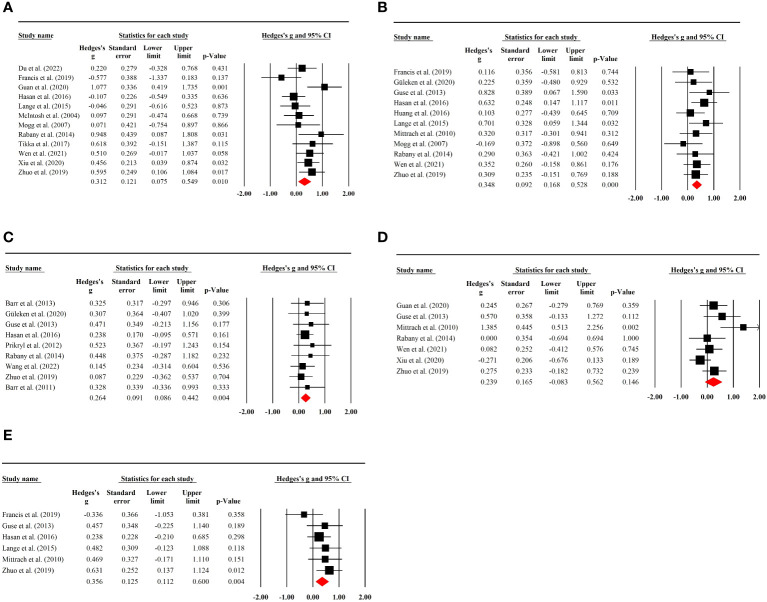
**(A)** Placebo effects of rTMS on memory in patients with SSD. **(B)** Placebo effects of rTMS on executive functioning in patients with SSD. **(C)** Placebo effects of rTMS on working memory in patients with SSD. **(D)** Placebo effects of rTMS on attention in patients with SSD. **(E)** Placebo effects of rTMS on processing speed in patients with SSD.

A total of 12 RCTs with 293 (70 female) patients were included in the analysis. The results showed that there was a significant pooled placebo ES for memory after sham rTMS treatment (g=0.31; 95% CI: 0.08 to 0.55; *p=*0.01; I^2^ = 48.80%; **Figure 3A**). The placebo effect size did not show a substantial difference when an RCT with high risk of bias was excluded (g=0.28; 95% CI: 0.02 to 0.53; *p=*0.032; I^2^ = 49.85%; see **Supplementary Table S3**).

Subgroup analysis of sham stimulation methods indicated that rTMS with 180° position coil (g=0.43, *p=*0.021; **Supplementary Figure S4a**), but not with sham coil, 45°, or 90° position coil, had a significant placebo effect. Separate analysis for rTMS targeting approaches revealed that rTMS with MRI-neuronavigation system (g=0.65, *p<*0.001; **Supplementary Figure S4b**) showed a significant placebo effect (see **Table 2**). In addition, a larger placebo effect size was observed in studies of the efficacy of active rTMS over sham rTMS (g=0.40, *p=*0.002; **Supplementary Figure S4d**)

No evident association was found between placebo effects of sham rTMS and age, proportion of females, illness duration, treatment days, sample size, randomization ratio, and the publication date (see **Supplementary Table S5**).

### 3.6 Placebo effects of rTMS on executive function

Executive function was reported in 11 RCTs involving 231 (60 female) patients. Sham rTMS had a significant placebo effect on executive function without study heterogeneity (g=0.35; 95% CI: 0.17 to 0.53; *p<*0.001; I^2^ = 0.00%; **Figure 3B**). No significant change in effect size was observed after excluding an RCT with high risk of bias (g=0.36; 95% CI: 0.16 to 0.55; *p<*0.001; I^2^ = 0.00%; **Supplementary Table S3**).

Subgroup analysis showed that sham rTMS with 45° or 90° position coil had an evident placebo effect on executive function (g=0.59, *p=*0.003; g=0.52, *p=*0.011; **Supplementary Figure S5a**), with the highest effect size found for the 45° position coil. The analysis also indicated that placebo effects of sham rTMS with 10–20 EEG location system or frequency of 10 Hz had a significant placebo effect (g=0.58, *p<*0.001; g=0.43, *p<*0.001; **Supplementary Figures S5b, c**) (see **Table 2**).

Meta-regression analysis revealed that placebo effects of sham rTMS on executive function was not affected by age, proportion of females, illness duration, treatment duration, sample size, randomization ratio, and publication time (see **Supplementary Table S5**).

### 3.7 Placebo effects of rTMS on working memory

Working memory tests were performed in nine rTMS RCTs containing 432 (126 female) patients. A significant placebo effect was found with no study heterogeneity (g=0.26; 95% CI:0.09 to 0.44; *p=*0.004; I^2^ = 0.00%; **Figure 3C**). Sensitivity analysis excluding two studies with high risk of bias showed a stable result (g=0.33; 95% CI: 0.12 to 0.54; *p<*0.01; I^2^ = 0.00%; **Supplementary Table S3**).

Subgroup analysis showed no significant placebo effects in the different sham stimulation methods, but a significant placebo effect of the rTMS protocol with 10–20 EEG location system (g=0.26, *p=*0.034; **Supplementary Figure S6b**) or 10-Hz stimulation frequency (g=0.32, *p=*0.024; **Supplementary Figure S6c**). Moreover, a significant placebo effect was found in studies where the efficacy of active rTMS was not superior to the sham rTMS (g=0.26, *p=*0.014; **Supplementary Figure S6d**)

### 3.8 Placebo effects of rTMS on attention

Seven RCTs with 375 (64 female) patients were used to analyze placebo effects on attention, and no significant placebo effect was observed (g=0.24; 95% CI: -0.08 to 0.56; *p=*0.146; I^2^ = 56.86%; **Figure 3D**). The result was stable when a study with a high risk of bias was excluded (g=0.25; 95% CI: -0.14 to 0.64; *p=*0.216; I^2^ = 63.26%; **Supplementary Table S3**). Additionally, there was no significant placebo effect found in the subgroup analysis (see **Table 2**).

### 3.9 Placebo effects of rTMS on processing speed

Six RCTs with 324 (74 female) patients were included in the analysis of placebo effects on processing speed. A significant placebo effect was found with a low degree of heterogeneity (g=0.36; 95% CI: 0.11 to 0.60; *p=*0.004; I^2^ = 7.63%; **Figure 3E**). Sensitivity analysis by removing an RCT with a high risk of bias showed no significant difference in the result (g=0.28; 95% CI: 0.01 to 0.54; *p=*0.039; I^2^ = 0.00%; **Supplementary Table S3**).

Subgroup analysis showed that sham rTMS with 10–20 EEG location system (g=0.44 *p=*0.001; **Supplementary Figure S8b**) or 10 Hz stimulation frequency (g=0.38, *p=*0.010; **Supplementary Figure S8c**) had a significant placebo effect (also see **Table 2**).

### 3.10 Sensitivity analysis

Apart from excluding studies with a high risk of bias to examine the reliability of the results, as described above for the relevant outcomes, we also estimated the placebo effect sizes assuming different pre-and post- treatment correlations (r=0, 0.5, and 0.8 respectively). As shown in **Supplementary Table S4**, little variation was observed in the sensitivity estimates. Specifically, the highest and lowest pooled placebo effect sizes were 0.44 and 0.43 for negative symptoms, 0.31 and 0.31 for memory, 0.35 and 0.30 for executive function, 0.26 and 0.26 for working memory, 0.23 and 0.19 for attention, and 0.37 and 0.30 for processing speed.

## 4 Discussion

This is the first systematic review of RCTs comprehensively investigating placebo effects of rTMS on negative symptoms and cognition in SSD. We found significant small-to-moderate placebo effect sizes in negative symptoms (g=0.44), memory (g=0.31), executive function (g=0.35), working memory (g=0.26) and processing speed (g=0.36) with low study heterogeneity and publication bias. However, no significant placebo effects of rTMS on attention were observed. The placebo effect size estimate was robust for each outcome in sensitivity analysis. Moreover, we identified several factors affecting placebo effects, including sham stimulation methods, rTMS targeting approaches, and rTMS frequency.

### 4.1 Placebo effect size in SSD

A meta-analysis by Fraguas et al. (79) was the first to examine placebo effects of pharmacological placebo on alleviating negative symptoms in SSD and found a significant placebo effect size of 2.909 (Cohen’s d). Based on this report, a study in 2022 re-evaluated placebo effects of antipsychotics on negative symptoms and found only a moderate effect size of 0.644 (Cohen’s d) (30). The reason for this difference between the two trials may be related to the different way the placebo effect size was calculated. Specifically, Fraguas et al. (79) calculated the placebo effect size using the mean change in negative symptoms over the follow-up period in the placebo group. Instead, Czobor et al. (30) calculate the placebo effect size by the mean change in negative symptoms between pre- and post-treatment measurements in the sham group. The methodology proposed by Czobor et al. is consistent with that employed in our study. Additionally, Keefe et al. (80) first investigated placebo effects of antipsychotic medications on cognition in SSD and found that the placebo treatment had minimal placebo effects with an effect size of 0.18 (Cohen’s d). Meanwhile, Agid et al. (81) reported a small to medium placebo effect size of about 0.33 for antipsychotics on total symptom severity in patients with SSD. It is matter to note that there is heterogeneity in the inclusion criteria of studies in these studies, and caution should be exercised when making comparisons.

In addition to pharmacological studies, non-invasive stimulation techniques such as rTMS have been considered as a promising method for alleviating symptoms of SSD. However, studies have shown that non-invasive techniques may produce greater placebo effects than pharmacological placebo (82, 83). Brunoni et al. (20) compared placebo effects of sham rTMS and pharmacological placebo in depression and found large placebo effects for both sham rTMS and pharmacological placebo. Dollfus and colleagues (23) found a placebo effect size of 0.29 using Hedges’ g in rTMS trials for the treatment of hallucinations, which is lower than the placebo effect sizes of rTMS in negative symptoms and several neurocognitive domains in this study. The subgroup analysis in their review showed a significant placebo effect in the parallel design RCTs (g=0.44), but not in the crossover trials. The lack of a placebo effect in crossover trials may be related to the fact that patients can differentiate between the active and the sham intervention periods in terms of scalp and auditory sensations, as well as coil placement on the head. Because the crossover design allows patients to compare the scalp and auditory sensations and side effects related to both active and sham stimulation periods, it is relatively easy for patients to guess which type of stimulation has been used. Therefore, awareness of the type of intervention may attenuate placebo effects in crossover trials. Apart from the fact that placebo effects of rTMS are present in SSD, studies have also reported that placebo effects are common in other psychiatric disorders, particularly in depression. A meta-analysis conducted by Xu et al. (84) reported that placebo effects of rTMS for depression were large (Cohen’s d = 1.016), and increasing over the years. Similarly, Razza et al. (17) analyzed 61 RCTs and also found that placebo effects in rTMS depression trials were large (Hedges’s g = 0.8) and positively associated with the year of publication. It should therefore be noted that placebo effects may be a common phenomenon in psychiatric conditions.

### 4.2 Predictors of placebo effects in SSD

In rTMS trials for psychiatric disorders, stimulation parameters are considered as important factors affecting the efficacy of rTMS (85, 86). Meanwhile, the parameters also influence placebo effects of sham rTMS, a view that has been reported in previous studies (20, 23). In our study, we found that sham stimulation methods, coil type, rTMS targeting approaches, and stimulation frequency and intensity influenced placebo effects to varying degrees.

Specifically, our findings indicated that the sham coil produced a significant placebo effect, which higher than that of other three sham stimulation methods in negative symptoms. The ideal sham coil would generally have the same appearance as the active coil. It can generate an identical stimulation sound and scalp sensation, but produces no or only a weak magnetic field (87), which does not induce an active effect on the cortical target. However, it is difficult to guarantee the quality of the sham coils applied in various studies, and therefore the effectiveness of blinding varies. Out of the 44 studies included, only 10 evaluated the effectiveness of blinding using a scale. All 10 studies reported that blinding was effective, as patients were unable to distinguish between the active and sham rTMS treatment. It should be noted that the stimulation sound and scalp sensation generated by sham coils with poor quality are clearly different from the active coils (87). It can therefore be easily distinguished from the active treatment by patients, which would impact the effectiveness of blinding. Moreover, studies have found that poorly designed sham coils can produce active effects, such as biological effects in the brain (88). Therefore, minimizing placebo effects by improving the design of sham coils will facilitate the development of rTMS techniques and provide more reliable evidence.

Moreover, we found that only the 180° or 45° position coil produced significant placebo effects on memory and executive function. Apart from the sham coil, it is also a common method of sham rTMS that an active coil is tilted at specific angles (e.g., 45°, 90°, and 180°) away from the scalp. Although this method is widely used, it has several drawbacks. First, the stimulation sensations in the scalp caused by the active coil tilted at a specific angle are not the same as those generated by the active coil. It can therefore easily lead subjects who have never received rTMS to believe that these feelings are side effects caused by the active treatment. Second, rTMS-induced somatic sensations are useful in increasing placebo effects (89). Meanwhile, the active coil angled away from the scalp has also been shown to produce the active effects in animal studies (24). Therefore, the positive effects of this sham condition may exaggerate the improvement in the clinical outcomes in the sham group. For the remaining cognitive domains, we found no significant placebo effects in any of sham stimulation methods, which may be related to the limited number of included studies. In addition to the aforementioned sham methods, it seems that transcutaneous electrical nerve stimulation (TENS) with a mild current may also be a viable option for simulating active rTMS as a sham control. This method is typically used to simulate the scalp sensation elicited by active rTMS without inducing real neuromodulatory effects. However, TENS was not employed as a sham control in the studies analyzed in our review. In a study by Sheffer et al. (90), it was reported that focal electrical stimulation could serve as an effective sham control for administering high-frequency rTMS at the dorsolateral prefrontal cortex. More RCTs are required to investigate and confirm the mimetic effect of TENS in rTMS studies.

There are currently three main approaches to locating the stimulation target in rTMS studies, including traditional anatomical localization (e.g. 5-cm rule), the 10–20 EEG location system, and the MRI-neuronavigation system (86). In our review, these methods were applied in 18, 11, and 11 studies, respectively. For negative symptoms, the method of anatomical localization was shown to produce the largest placebo effect and the MRI-neuronavigation system the smallest. For memory, a significant placebo effect was only observed in the method of MRI-neuronavigation system. For working memory, attention, and processing speed, only the 10–20 EEG location system shown a significant placebo effect. It has been suggested that when physicians use more advanced technology, particularly the MRI-neuronavigation system, patients are prefer to assume that they are receiving effective treatment and therefore develop good expectations of treatment (23). Meanwhile, other two methods also require careful manipulation by professionals, so their placebo effects may depend heavily on the performance of the professionals. However, it is not yet known whether these effects are real and how large they are. Overall, we need to be problem specific, and that different rTMS targeting approaches may cause the inconsistent size of placebo effects across symptoms.

In general, the duration of an rTMS session increases as the rTMS frequency decreases. Longer stimulation duration may increase the opportunity for patient-doctor interaction and enhance placebo effects. Our results indicate that a stimulation frequency of 10 Hz could induce significant placebo effects in negative symptoms, executive function, working memory, and processing speed. However, the 20 Hz stimulation frequency only produces significant placebo effects for negative symptoms. It is important to note that due to the limited number of studies using 5 Hz and 50 Hz stimulation in our review, placebo effect sizes are missing for all cognitive domains, making it inappropriate to compare them with 10 Hz and 20 Hz for placebo effects. The relationship between stimulation frequency and placebo effects still needs to be investigated due to the limited number of studies analyzed in this review. Moreover, The number of intervention sessions may be positively correlated with the study duration. In our review, the majority of intervention sessions in the included studies ranged from 10 to 40 sessions. Although there is a paucity of evidence demonstrating the relationship between placebo effects and intervention sessions, it is possible that they may play a role in influencing the effects. Thus, further research is necessary to examine the potential relationship.

Publication time and subject demographics have previously been considered as potential factors influencing placebo effects (91). Our results suggest that there were no significant placebo effects of these factors on negative symptoms and cognition in SSD. It is therefore still debated whether factors, such as age, sex, and proportion of females affect placebo effects in psychiatry (92). In a study by Czobor et al. (30), they found that placebo effects were significantly related to study duration. Specifically, it decreased over the 8-week study period. In our included studies, the study duration was mostly between 2 and 4 weeks, with relatively few studies lasting 8 weeks or more. This may be the reason why we did not find a significant association between study duration and placebo effects. In addition, placebo effects may increase over the years of the study (93), but this phenomenon was not found in our study and in the study by Czobor et al. as well. Regarding the relationship between illness duration and placebo effects, although no significant association was found, a negative association has been reported in previous researches (30, 81). Several studies have shown that unbalanced randomization could also increase placebo effects (30, 94), and this association was not significant in our study. Apart from the above factors, several subjective factors also need to be considered. First, previous studies have reported that patients’ expectations and their relationship with the clinician influence placebo effects during treatment (95, 96). Patient expectations are the beliefs that patients hold about the potential benefits of the treatment they receive. Researches indicated that patient expectations are associated with the secretion of dopamine, altered neuronal firing, or changes in brain glucose metabolism (97). The rational promotion of patient expectations in medical practice can help to maximize the efficacy of rTMS, improving the treatment of psychiatric disorders (84). With the increasing recognition of physiotherapy and advancements in psychiatry, rTMS for SSD is gaining wider acceptance among the general public (98). This may result in a sustained increase in placebo effects, and potentially increasing its significance in clinical settings. Second, healthcare professionals’ competence and empathy are also associated with placebo effects (99). It is therefore advisable for researchers to control for the potential effects of those subjective factors to reduce the bias. In medical practice, investigating placebo effects and the moderators may assist clinicians, in comprehending the underlying mechanisms and in developing more effective treatment strategies. It can also facilitate the reduction of placebo effects through proper methods, thereby increasing sensitivity to detect effects of promising rTMS protocols for improving negative symptoms and cognitive impairments in clinical settings.

With regard to the possible neurobiological mechanisms underlying placebo effects, some evidence suggests that it may be related to the release of dopamine in the mesolimbic pathway (100). Dopamine has been shown to activate endogenous reward networks in the brain, including the ventromedial prefrontal cortex, ventral striatum, orbitofrontal cortex, anterior cingulate cortex, and amygdala, leading to clinical benefits. It should also be noted that the mechanisms are complex and not yet well understood, and more research is required.

The findings of the current review should be considered with several limitations. First, although subgroup analysis was performed for each outcome to explore placebo effects under the different conditions, the limited number of studies in some subgroups resulted in missing results. Second, due to the insufficient information in the included RCTs, we are not able to examine all factors that may affect placebo effects in SSD such as study design (parallel and cross-over) and number of research centers. Finally, we did not examine placebo effects of rTMS on social cognition, as the very limited publications.

## 5 Conclusion

Overall, our study provides up-to-date evidence of placebo effects of rTMS on negative symptoms and cognitive impairment in SSD. We conclude that placebo effects of rTMS on negative symptoms, memory, executive function, working memory, processing speed, but not on attention, are significant in a small-to-moderate magnitude. Subgroup analysis and meta-regressions showed that placebo effects were significantly associated with sham stimulation methods, rTMS targeting approaches, and stimulation frequency, but not with age, proportion of females, illness or treatment duration, sample size, randomization proportion and publication time. Our findings, therefore, provide novel insights into understanding placebo effects of rTMS on negative symptoms and cognitive deficits, which would accelerate the development of sham-controlled rTMS studies in the treatment of SSD. More well-designed RCTs with larger sample sizes and longer follow-up may be needed to investigate novel rTMS protocols to minimize placebo effects in the treatment of negative and cognitive symptoms in SSD.

## Data availability statement

The original contributions presented in the study are included in the article/**Supplementary Material**. Further inquiries can be directed to the corresponding authors.

## Author contributions

MW: Conceptualization, Data curation, Methodology, Writing – original draft. SL: Data curation, Software, Writing – original draft. LH: Data curation, Writing – review & editing. YX: Software, Writing – review & editing. ZS: Software, Supervision, Writing – review & editing. LS: Conceptualization, Supervision, Writing – review & editing.
