# Supplementary material for: Placebo effects of repetitive transcranial magnetic stimulation on negative symptoms and cognition in patients with schizophrenia spectrum disorders: a systematic review and meta-analysis
Source: Front Psychiatry. 2024 May 28;15:1377257. doi: 10.3389/fpsyt.2024.1377257 (PMC11165700; doi:10.3389/fpsyt.2024.1377257)
Supplement: Supplementary file 1 [file DataSheet_1.docx]

Supplementary Material

**Supplementary Table 1.** Search strategy.

**Supplementary Table 2.** Measures used to assess negative symptoms and cognition in the included studies.

**Supplementary Table 3.** Sensitivity analyses by removing studies with high risk of bias.

**Supplementary Table 4.** Overall placebo effect sizes estimate from sensitivity analyses.

**Supplementary Table 5.** Coefficients of predictors for rTMS trials of placebo effects in negative symptoms and cognition.

**Supplementary Figure 1a-c.** Funnel plots for publications investigating the placebo effects of rTMS on negative symptoms, memory, and executive function.

**Supplementary Figure 2a-b.** The risk of bias assessments for randomized designed trials with parallel and cross-over design.

**Supplementary Figure 3a-d** Forest plots showing subgroup analyses for placebo effects of rTMS on negative symptoms.

**Supplementary Figure 4a-d** Forest plots showing subgroup analyses for placebo effects of rTMS on memory.

**Supplementary Figure 5a-c** Forest plots showing subgroup analyses for placebo effects of rTMS on executive function.

**Supplementary Figure 6a-d** Forest plots showing subgroup analyses for placebo effects of rTMS on working memory.

**Supplementary Figure 7a-c** Forest plots showing subgroup analyses for placebo effects of rTMS on attention.

**Supplementary Figure 8a-c** Forest plots showing subgroup analyses for placebo effects of rTMS on processing speed.

**Full list of included studies**

**Table S1.** Search strategy

| Key words | |
| --- | --- |
| “cognition,” “cognitive,” “cognitive functioning,” “neurocognitive,” “attention,” “working memory,” “executive function,” “memory,” “learning,” “processing speed,” “negative symptoms,” “alogia,” “anhedonia,” “avolition,” “amotivation,” “asociality,” “blunted affect,” “non-invasive brain stimulation,” “NIBS,” “repetitive transcranial magnetic stimulation,” “rTMS,” “theta burst stimulation,” “TBS,” “iTBS,” “cTBS,” “schizophrenia,” “schizophrenic disorder,” “schizoaffective,” and “dementia praecox.” | |
| Search strings | |
| Medline  (PubMed) | (((cognition OR cognitive OR "cognitive functioning" OR neurocognitive OR attention OR "working memory" OR "executive function" OR memory OR learning OR "processing speed" OR "negative symptoms" OR alogia OR anhedonia OR avolition OR amotivation OR asociality OR "blunted affect")) AND ((rTMS OR "repetitive transcranial magnetic stimulation" OR "theta burst stimulation" OR TBS OR iTBS OR cTBS OR "non-invasive brain stimulation" OR NIBS))) AND ((schizophrenia OR "schizophrenic disorder" OR schizoaffective OR "dementia praecox")) |
| Web of Science | ((TS=(cognition OR cognitive OR "cognitive functioning" OR neurocognitive OR attention OR "working memory" OR "executive function" OR memory OR learning OR "processing speed" OR "negative symptoms" OR alogia OR anhedonia OR avolition OR amotivation OR asociality OR "blunted affect")) AND TS=(rTMS OR "repetitive transcranial magnetic stimulation" OR "theta burst stimulation" OR TBS OR iTBS OR cTBS OR "non-invasive brain stimulation" OR NIBS)) AND TS=(schizophrenia OR "schizophrenic disorder" OR schizoaffective OR "dementia praecox") |
| EMBASE (Ovid) | ((cognition or cognitive or "cognitive functioning" or neurocognitive or attention or "working memory" or "executive function" or memory or learning or "processing speed" or "negative symptoms" or alogia or anhedonia or avolition or amotivation or asociality or "blunted affect") and (rTMS or "repetitive transcranial magnetic stimulation" or "theta burst stimulation" or TBS or iTBS or cTBS or "non-invasive brain stimulation" or NIBS) and (schizophrenia or "schizophrenic disorder" or schizoaffective or "dementia praecox")).af. |
| Cochrane library | (cognition OR cognitive OR cognitive functioning OR neurocognitive OR attention OR working memory OR executive function OR memory OR learning OR processing speed OR negative symptoms OR alogia OR anhedonia OR avolition OR amotivation OR asociality OR blunted affect) in Title Abstract Keyword AND (rTMS OR repetitive transcranial magnetic stimulation OR theta burst stimulation OR TBS OR iTBS OR cTBS OR non-invasive brain stimulation OR NIBS) in Title Abstract Keyword AND (schizophrenia OR schizophrenic disorder OR schizoaffective OR dementia praecox) in Title Abstract Keyword - (Word variations have been searched) |
| CINAHL | ( (cognition OR cognitive OR "cognitive functioning" OR neurocognitive OR attention OR "working memory" OR "executive function" OR memory OR learning OR "processing speed" OR "negative symptoms" OR alogia OR anhedonia OR avolition OR amotivation OR asociality OR "blunted affect") ) AND ( (rTMS OR "repetitive transcranial magnetic stimulation" OR "theta burst stimulation" OR TBS OR iTBS OR cTBS OR "non-invasive brain stimulation" OR NIBS) ) AND ( (schizophrenia OR "schizophrenic disorder" OR schizoaffective OR "dementia praecox") ) English Language; Peer Reviewed; Research Article; Human AND Apply related words; Also search within the full text of the articles; |

**Table S2.** Measures used to assess negative symptoms and cognition in the included studies

| **Outcomes** | **Assessment instruments** |
| --- | --- |
| Negative symptoms | CAINS; PANSS; SANS. |
| Memory | BACS; CANTAB; Drawing Test; MCCB; RBANS; VLMT. |
| Executive function | BACS; CANTAB; COWAT; SCWT; TMT-B; WCST. |
| Working memory | CANTAB; DST; MCCB; N-back Task; VFT. |
| Attention/vigilance | BACS; CANTB; D2; MCCB; RBANS; TAP. |
| Processing speed | BACS; MCCB; TMT-A. |
| Abbreviations: BACS: Brief Assessment of Cognition in Schizophrenia; CANTAB: Cambridge Neuropsychological Test Automated Battery; CAINS: Clinical Assessment Interview for Negative Symptoms; COWAT: Controlled Oral Word Association Test; DST: Digit Span Test; D2: D2-Attention Task; MCCB: MATRICS Consensus Cognitive Battery; PANSS: Positive and Negative Syndrome Scale; RBANS: Repeatable Battery for the Assessment of Neuropsychological Status; SANS: Assessment of Negative Symptoms; SCWT: Stroop Color and Word Test; TMT-A=Trail Making Test Part A; TMT-B=Trail Making Test Part B; TAP: Tübinger Aufmerksamkeitsprüfung; VLMT: Verbal Learning and Memory Test; VFT: Verbal Fluency Test; WCST: Wisconsin Card Sorting Test. | |

**Table S3.** Sensitivity analyses by removing studies with high risk of bias

| **Outcomes** | **k** | **Hedge’s g** | **SE** | **Lower limit** | **Higher limit** | **p** | **I^2^ (%)^a^** | **I^2^ (%)^b^** |
| --- | --- | --- | --- | --- | --- | --- | --- | --- |
| Negative symptoms | 35 | 0.44 | 0.06 | 0.32 | 0.57 | 0.000 | 43.73 | 43.12 |
| Memory | 11 | 0.28 | 0.13 | 0.02 | 0.53 | 0.032 | 49.85 | 48.80 |
| Executive function | 10 | 0.36 | 0.10 | 0.16 | 0.55 | 0.000 | 0.00 | 0.00 |
| Working memory | 7 | 0.33 | 0.11 | 0.12 | 0.54 | 0.003 | 0.00 | 0.00 |
| Attention | 6 | 0.25 | 0.20 | -0.14 | 0.64 | 0.216 | 63.26 | 56.86 |
| Processing speed | 5 | 0.28 | 0.14 | 0.01 | 0.54 | 0.039 | 0.00 | 7.63 |

K: the number of included RCTs; SE: Standard error.

^a^: I^2^ for each outcome in sensitivity analyses; ^b^: I^2^ for each outcome in original analyses.

**Table S4.** Overall placebo effect sizes estimate from sensitivity analyses

| **Outcomes** | **Hedge' s g estimate from sensitivity analysis (r = 0)** | | **Hedge' s g estimate from sensitivity analysis (r = 0.5)** | | **Hedge' s g estimate from sensitivity analysis (r = 0.8)** | |
| --- | --- | --- | --- | --- | --- | --- |
|  | ES (SE) | p | ES (SE) | p | ES (SE) | p |
| Negative symptoms | 0.44  (0.06) | 0.000 | 0.44  (0.06) | 0.000 | 0.43  (0.06) | 0.000 |
| Memory | 0.31  (0.12) | 0.011 | 0.31  (0.12) | 0.009 | 0.31  (0.11) | 0.006 |
| Executive function | 0.35  (0.11) | 0.001 | 0.34  (0.08) | 0.000 | 0.30  (0.07) | 0.000 |
| Working memory | 0.26  (0.11) | 0.012 | 0.26  (0.07) | 0.000 | 0.26  (0.05) | 0.000 |
| Attention | 0.23  (0.17) | 0.180 | 0.23  (0.15) | 0.119 | 0.19  (0.12) | 0.107 |
| Processing speed | 0.37  (0.14) | 0.008 | 0.34  (0.12) | 0.006 | 0.30  (0.11) | 0.009 |

**Table S5.** Coefficients of predictors for rTMS trials of placebo effect in negative symptoms and cognition.

| **Moderator Variables** | **Negative symptoms** | **Memory** | **Executive function** |
| --- | --- | --- | --- |
|  | $\beta$ (p) | $\beta$ (p) | $\beta$ (p) |
| Mean age | -0.004 (0.567) | 0.019 (0.157) | 0.002 (0.924) |
| Female proportion | 0.005 (0.683) | -0.423 (0.609) | 0.367 (0.623) |
| Illness duration | -0.002 (0.826) | 0.018 (0.176) | 0.003 (0.829) |
| Treatment days | 0.012 (0.150) | 0.021 (0.104) | 0.003 (0.904) |
| Sample size | 0.004 (0.254) | 0.016 (0.285) | 0.009 (0.629) |
| Active vs. sham ratio | -0.079 (0.542) | 0.451 (0.187) | -0.268 (0.382) |
| Publication year | 0.005 (0.683) | 0.021 (0.592) | 0.013 (0.607) |

Note: Univariate Regression Analysis.


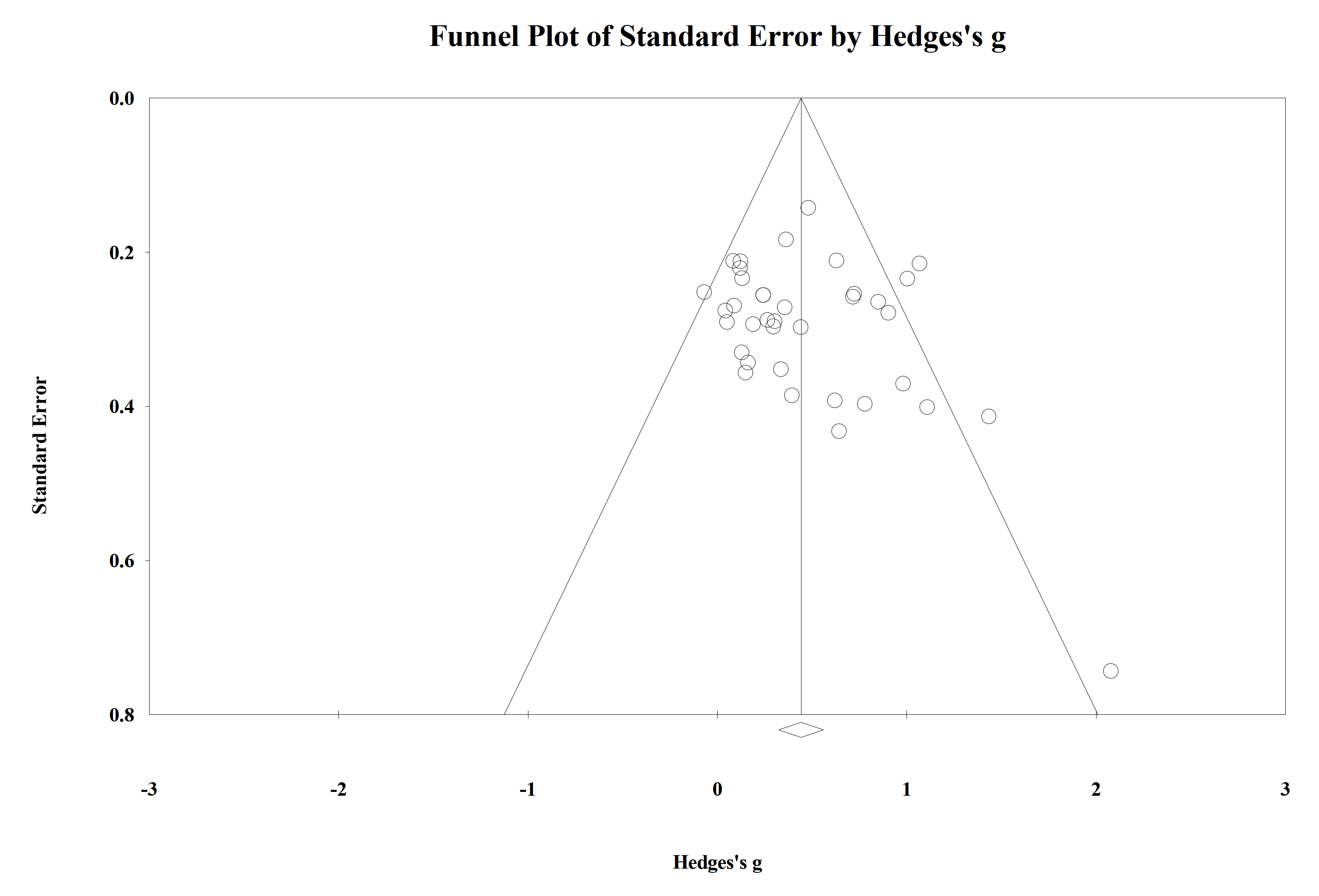


**Figure S1a.** Funnel plot for publications investigating the placebo effect of rTMS on negative symptoms.


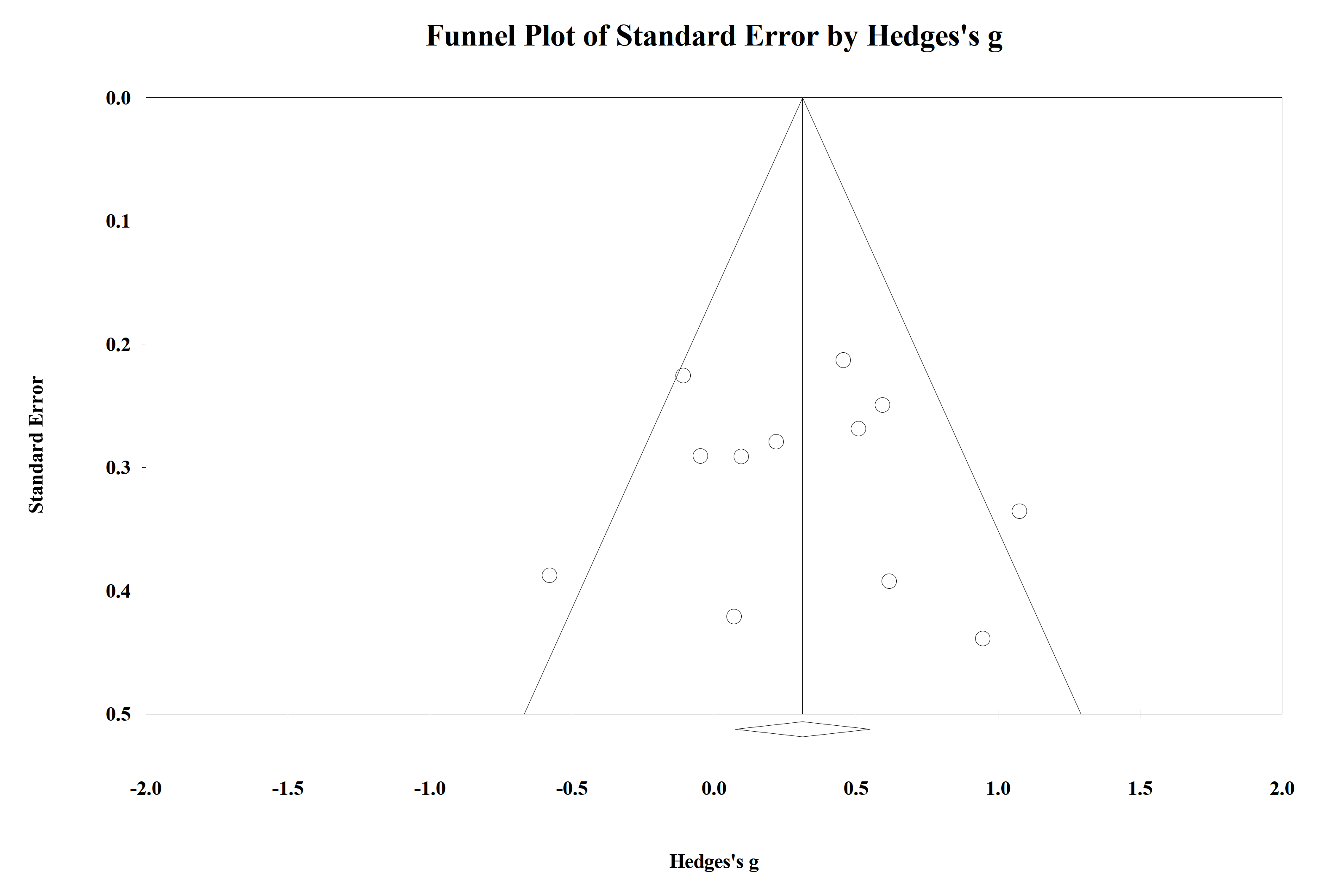


**Figure S1b.** Funnel plot for publications investigating the placebo effect of rTMS on memory.


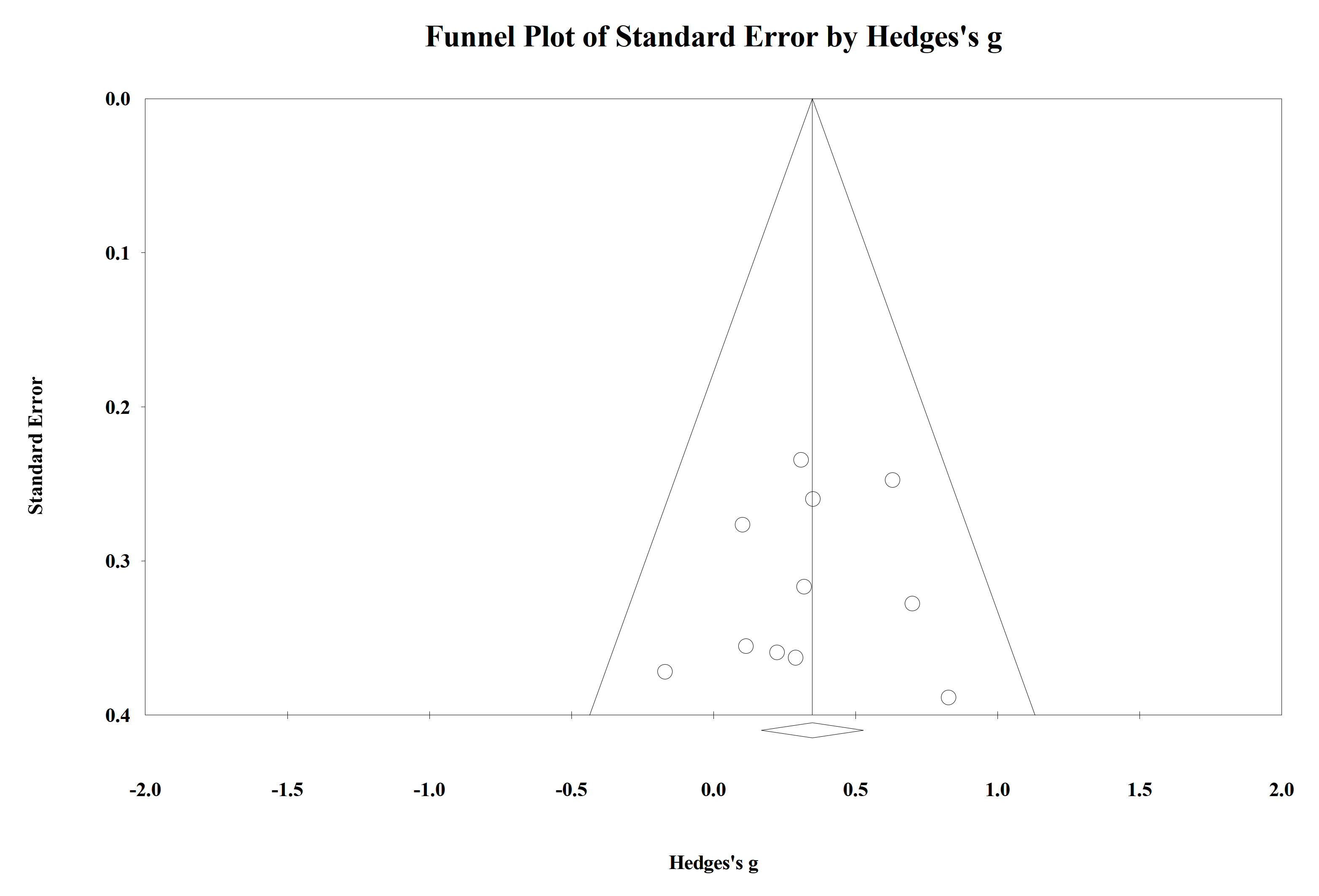


**Figure S1c.** Funnel plot for publications investigating the placebo effect of rTMS on executive function.

**Figure S2a.** The risk of bias assessments for randomized parallel-designed trials.

**Figure S2b.** The risk of bias assessment for randomized crossover-designed trial.

**Negative symptoms**


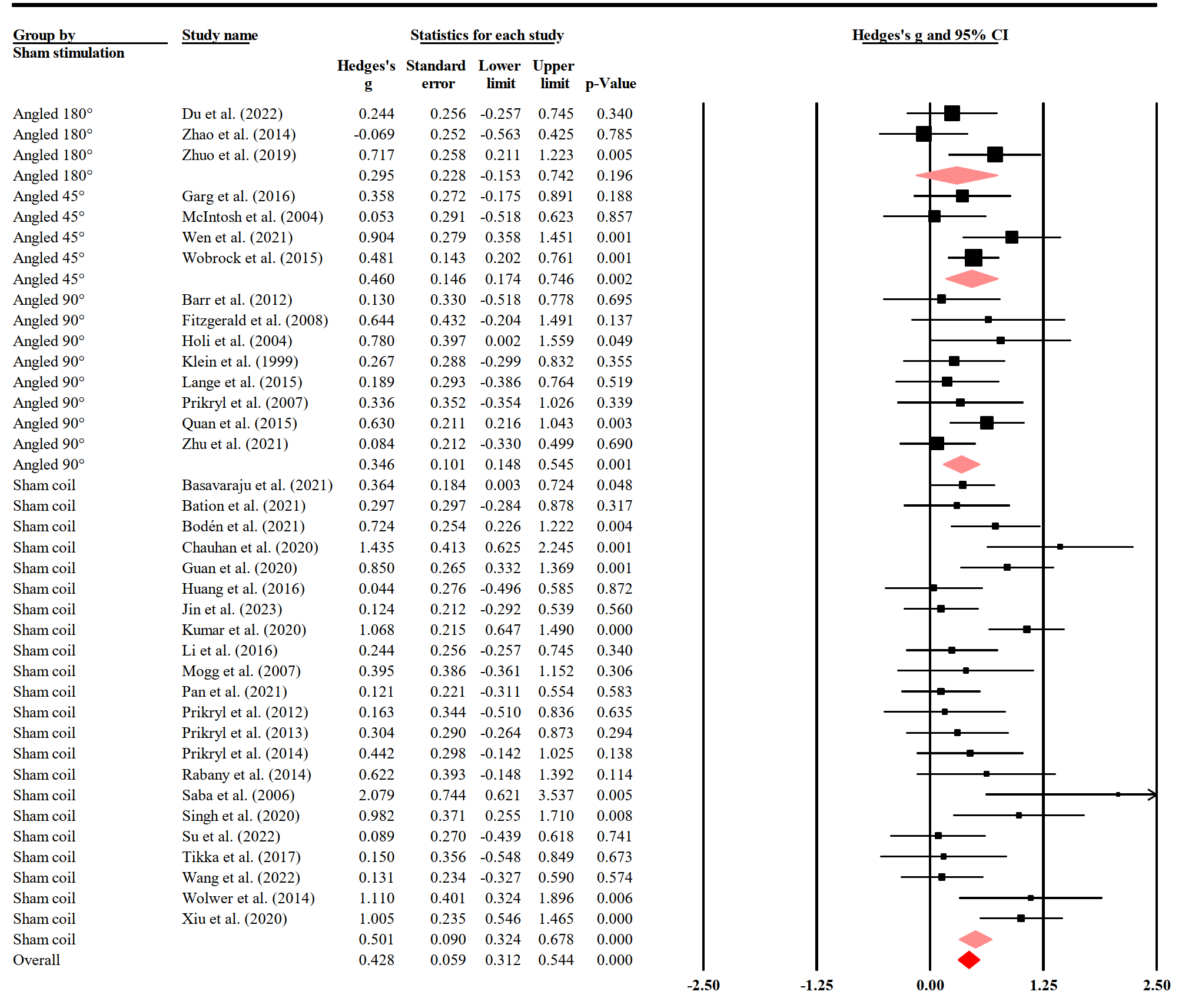


**Figure S3a.** Forest plot showing subgroup analyses grouped by sham conditions in negative symptoms.


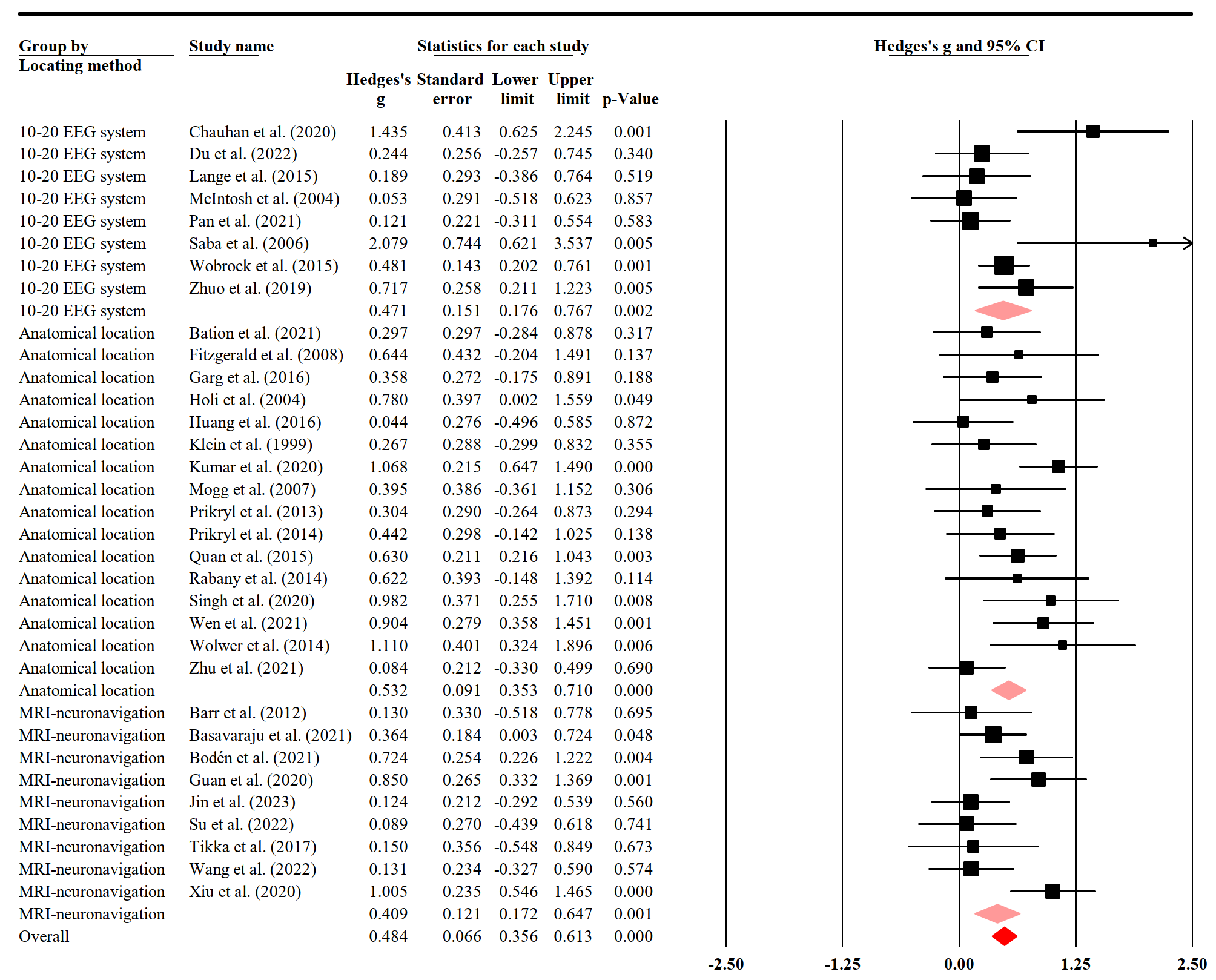


**Figure S3b.** Forest plot showing subgroup analyses for placebo effects grouped by methods of target localization in negative symptoms.


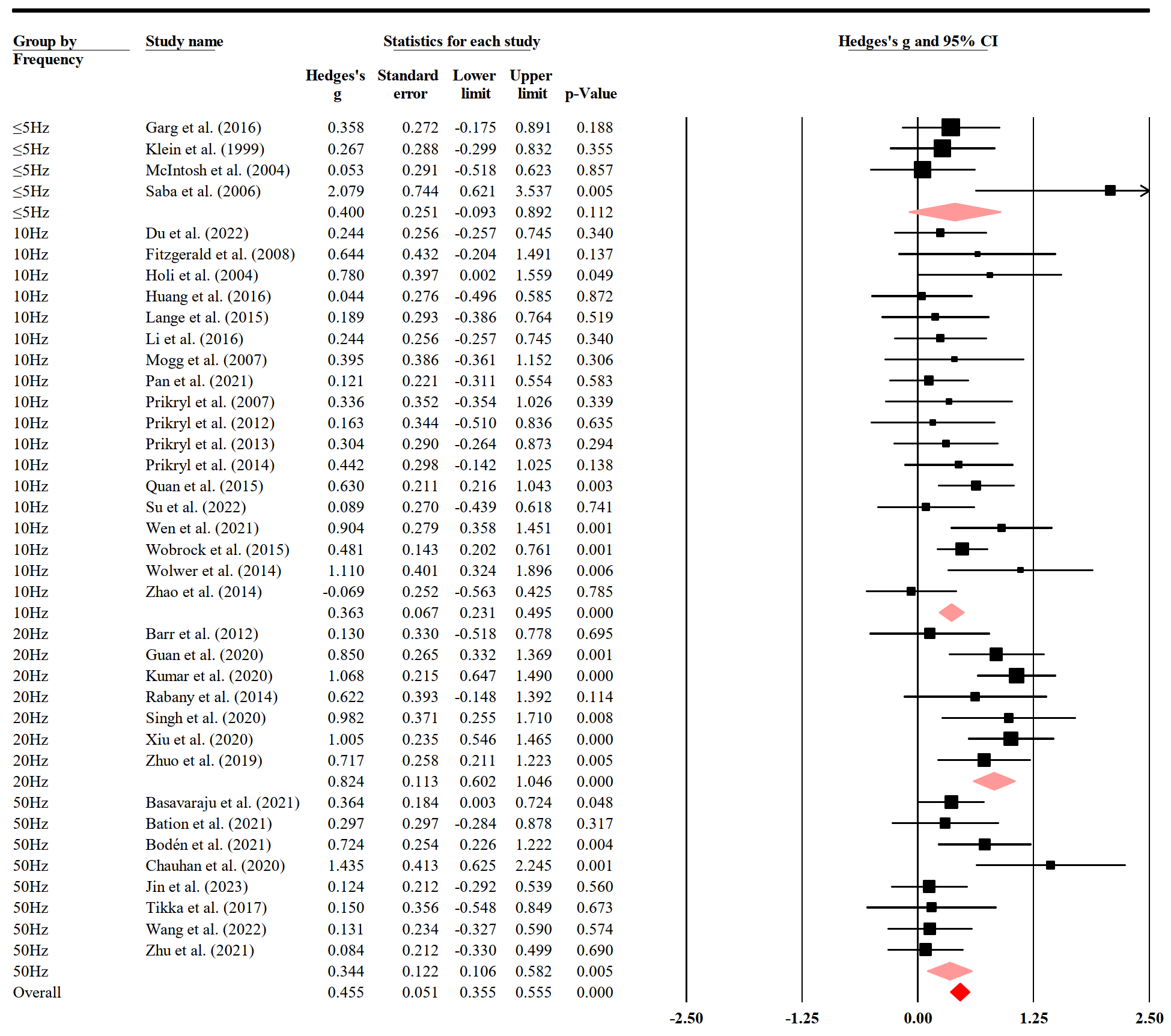


**Figure S3c.** Forest plot showing subgroup analyses for placebo effects by frequency in negative symptoms.


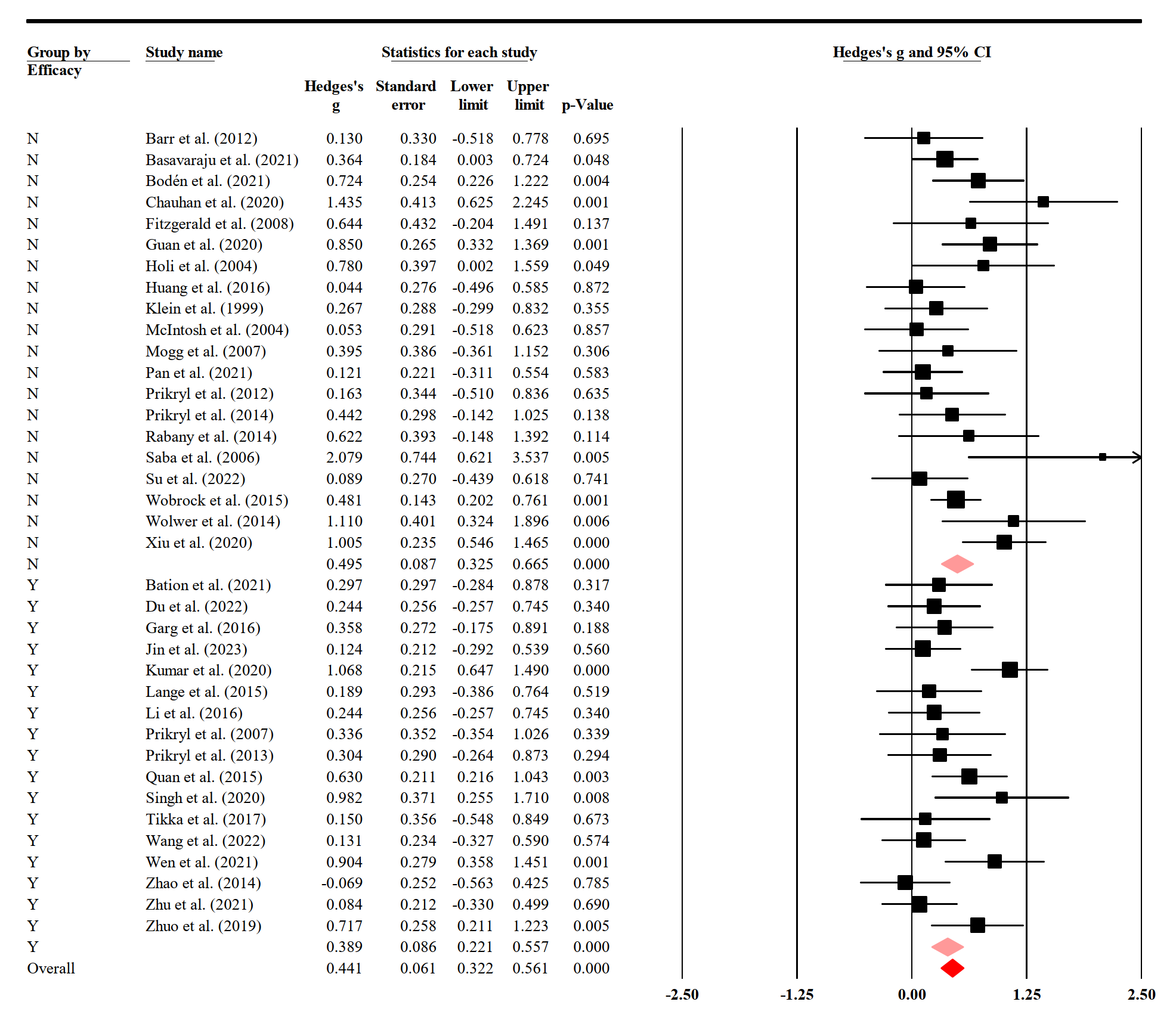


**Figure S3d.** Forest plot showing subgroup analyses for placebo effects grouped by efficacy of active rTMS over sham rTMS in negative symptoms.

**Memory**


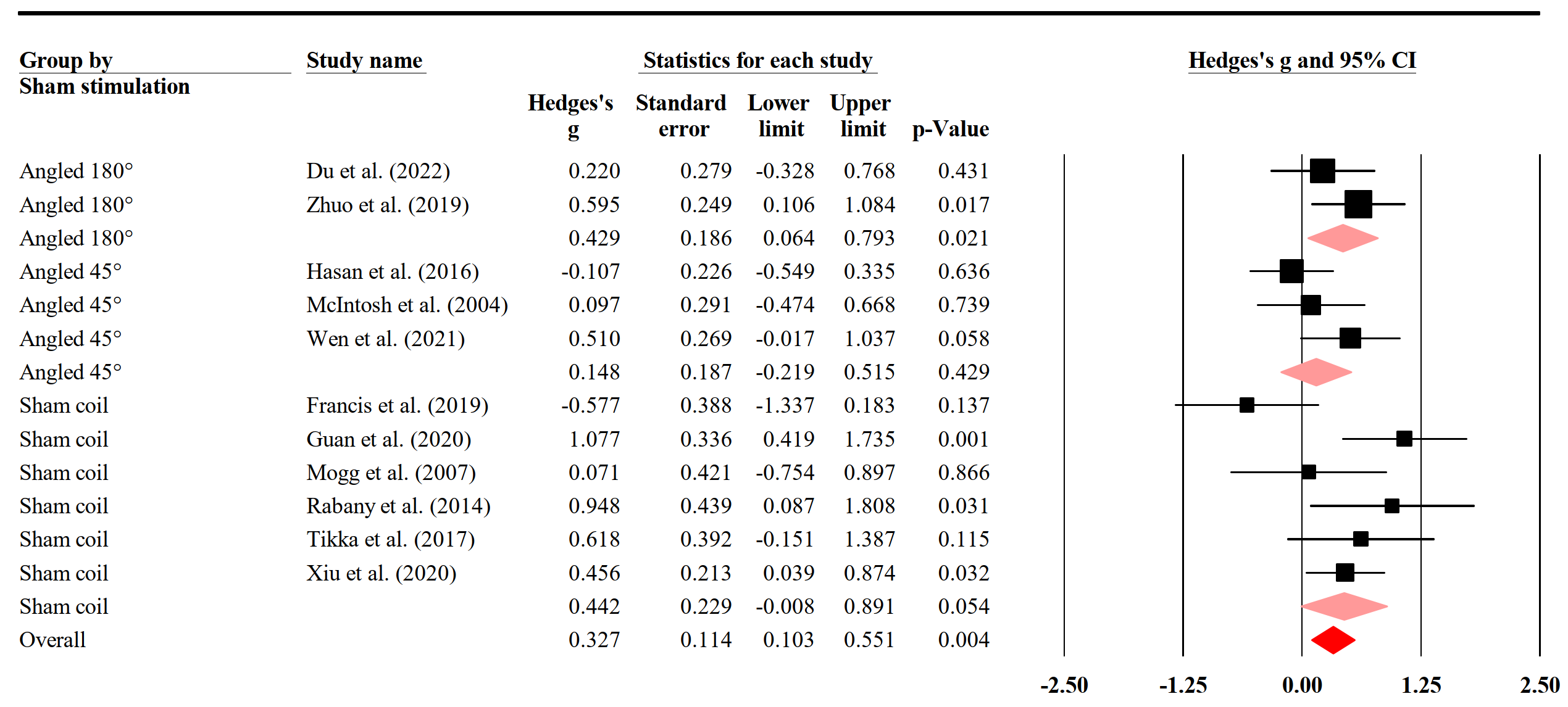


**Figure S4a.** Forest plot showing subgroup analyses for placebo effects grouped by sham conditions in memory.


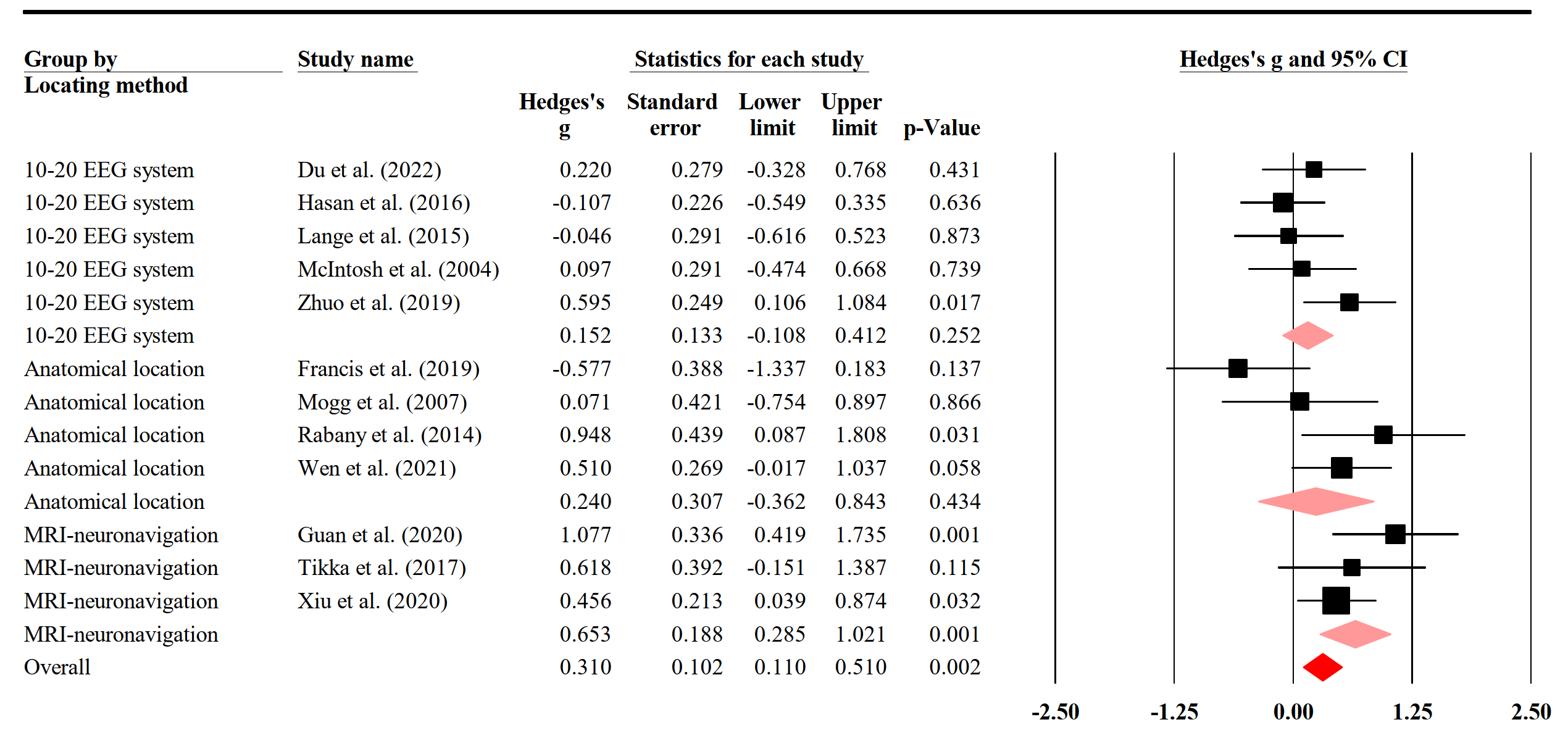


**Figure S4b.** Forest plot showing subgroup analyses for placebo effects grouped by methods of target localization in memory.


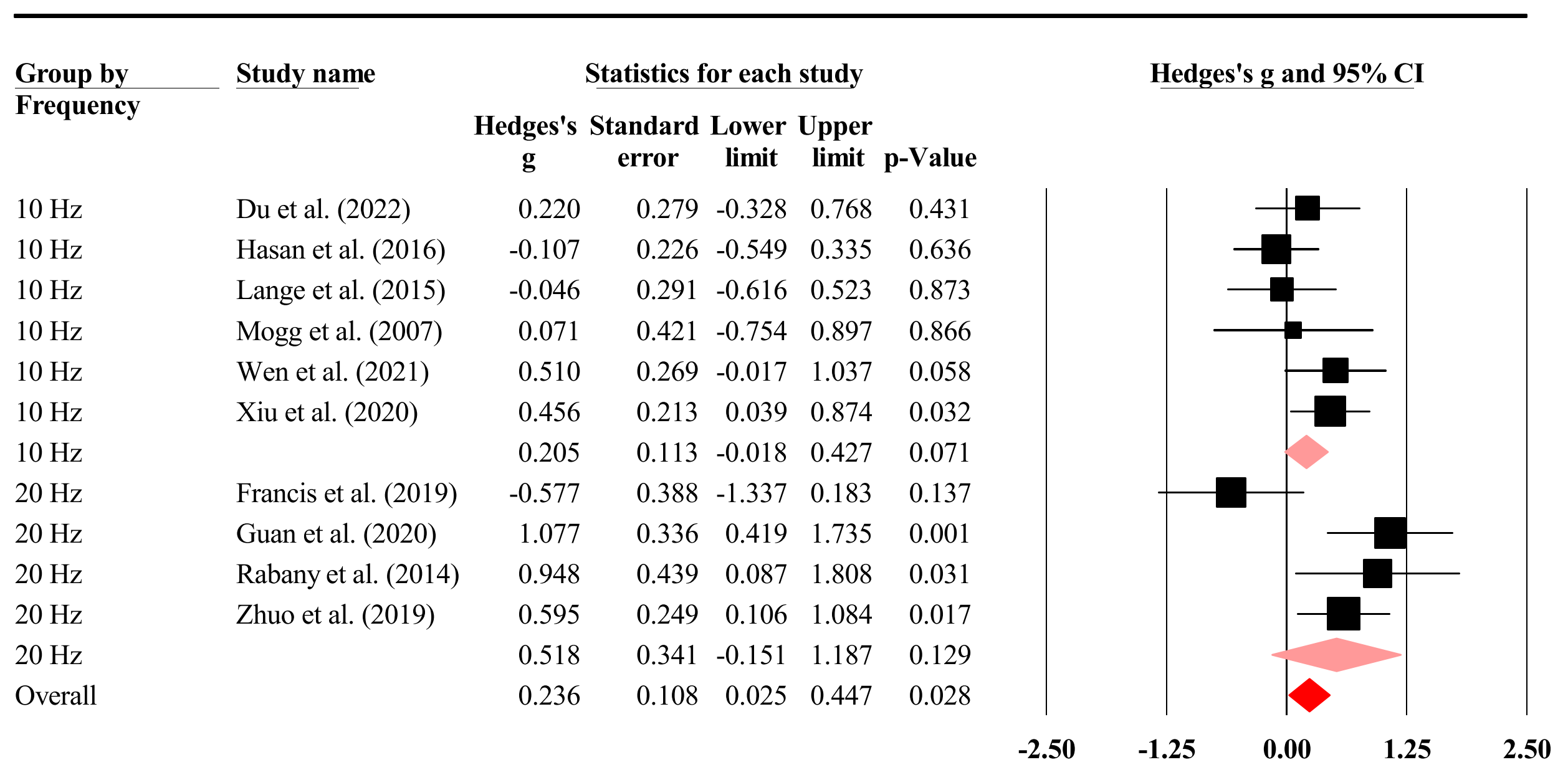


**Figure S4c.** Forest plot showing subgroup analyses for placebo effects grouped by frequency in memory.


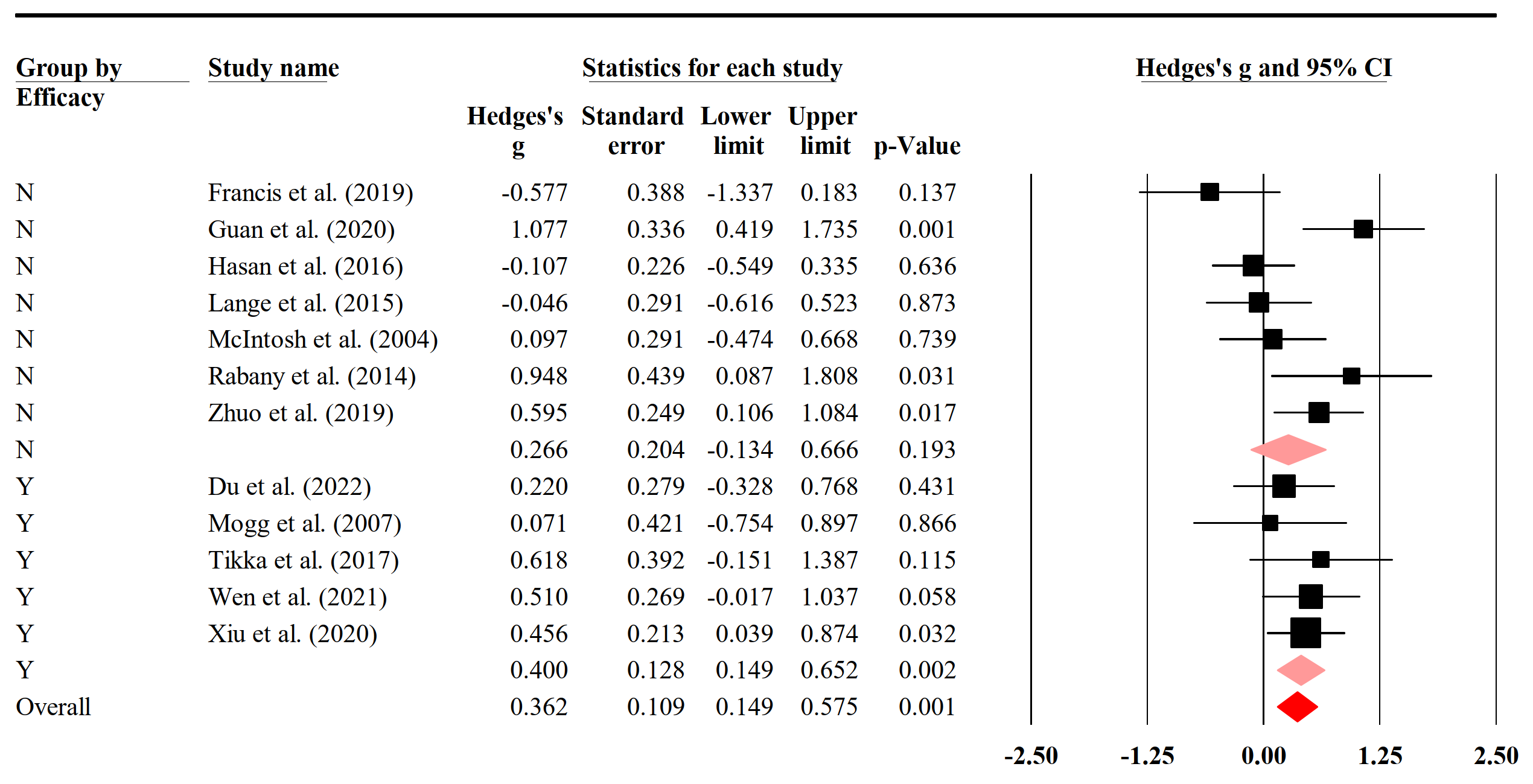


**Figure S4d.** Forest plot showing subgroup analyses for placebo effects grouped by efficacy of active rTMS over sham rTMS in memory.

**Executive function**


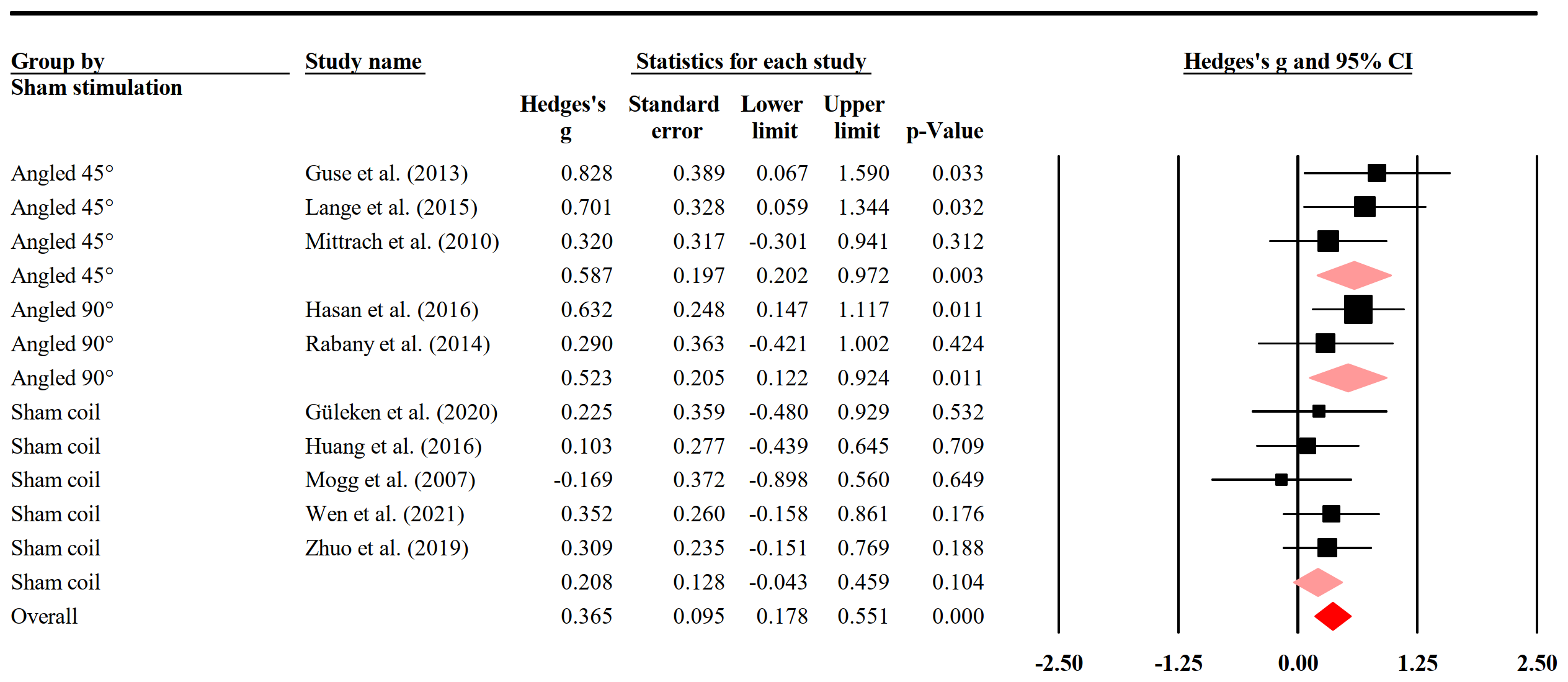


**Figure S5a.** Forest plot showing subgroup analyses for placebo effects grouped by sham conditions in executive function.


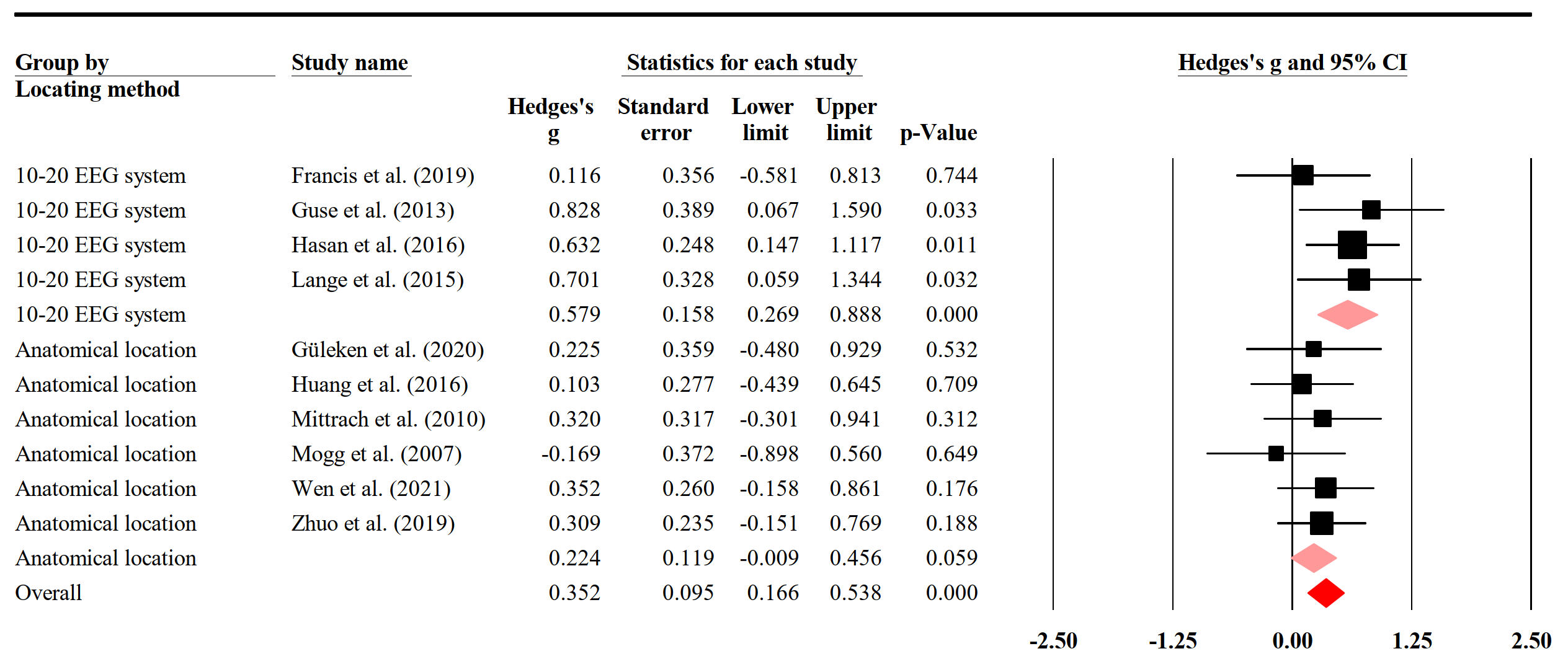


**Figure S5b.** Forest plot showing subgroup analyses for placebo effects grouped by methods of target localization in executive function.


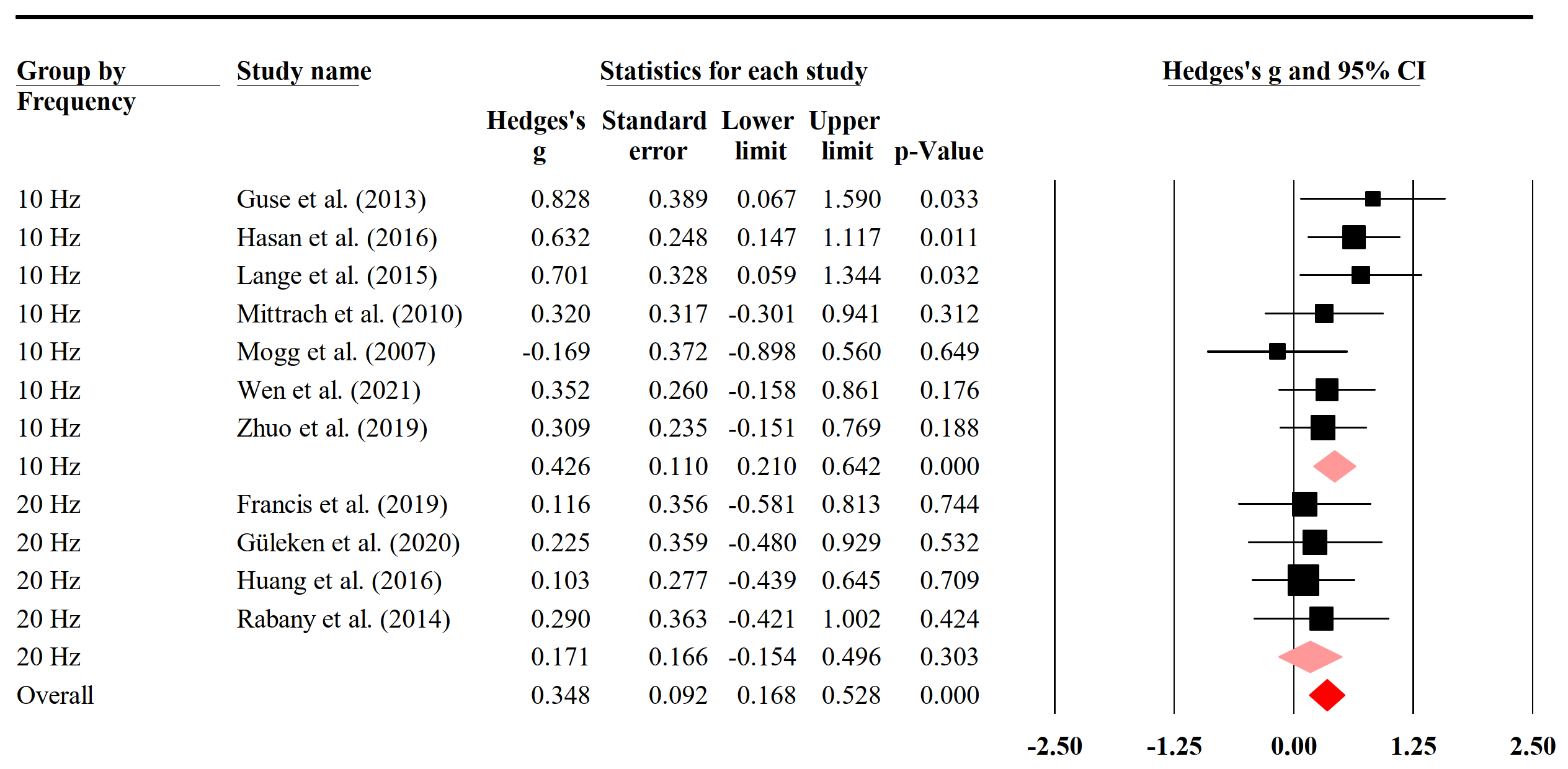


**Figure S5c.** Forest plot showing subgroup analyses for placebo effects grouped by frequency in executive function.

**Working memory**


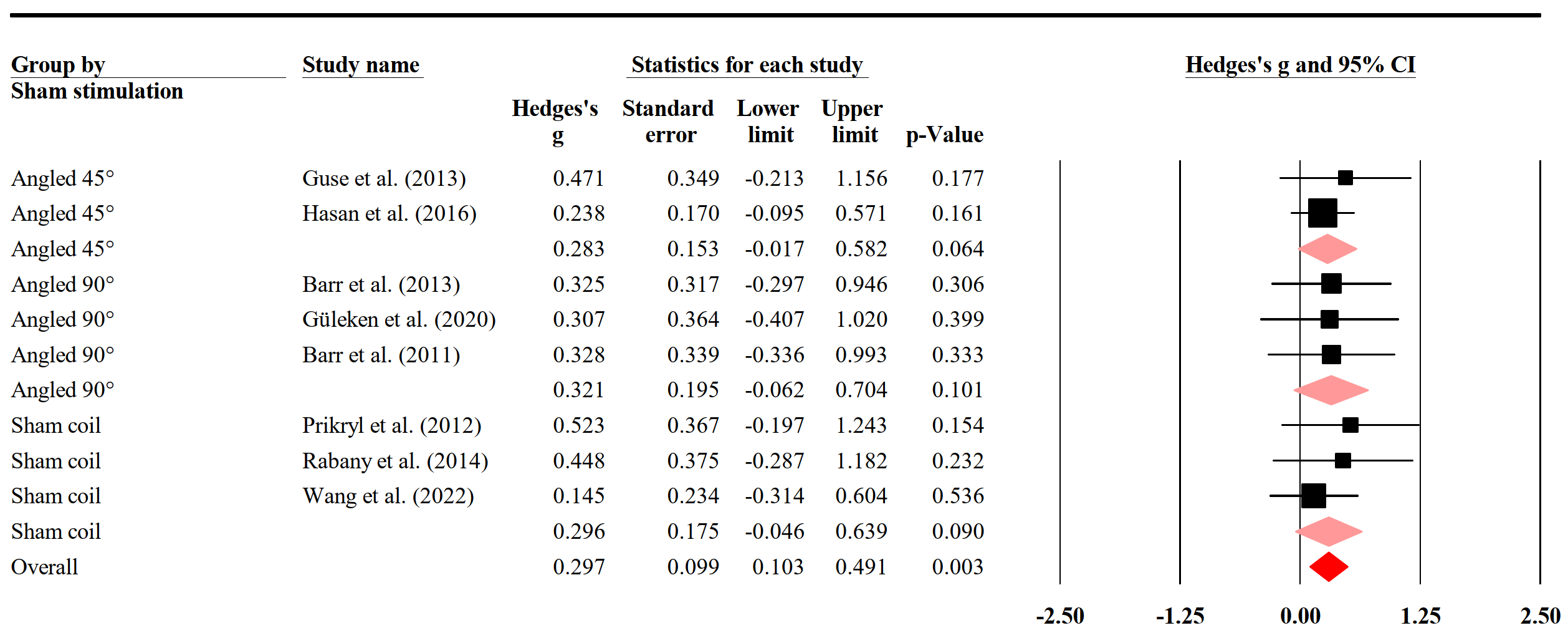


**Figure S6a.** Forest plot showing subgroup analyses for placebo effects grouped by sham conditions in working memory.


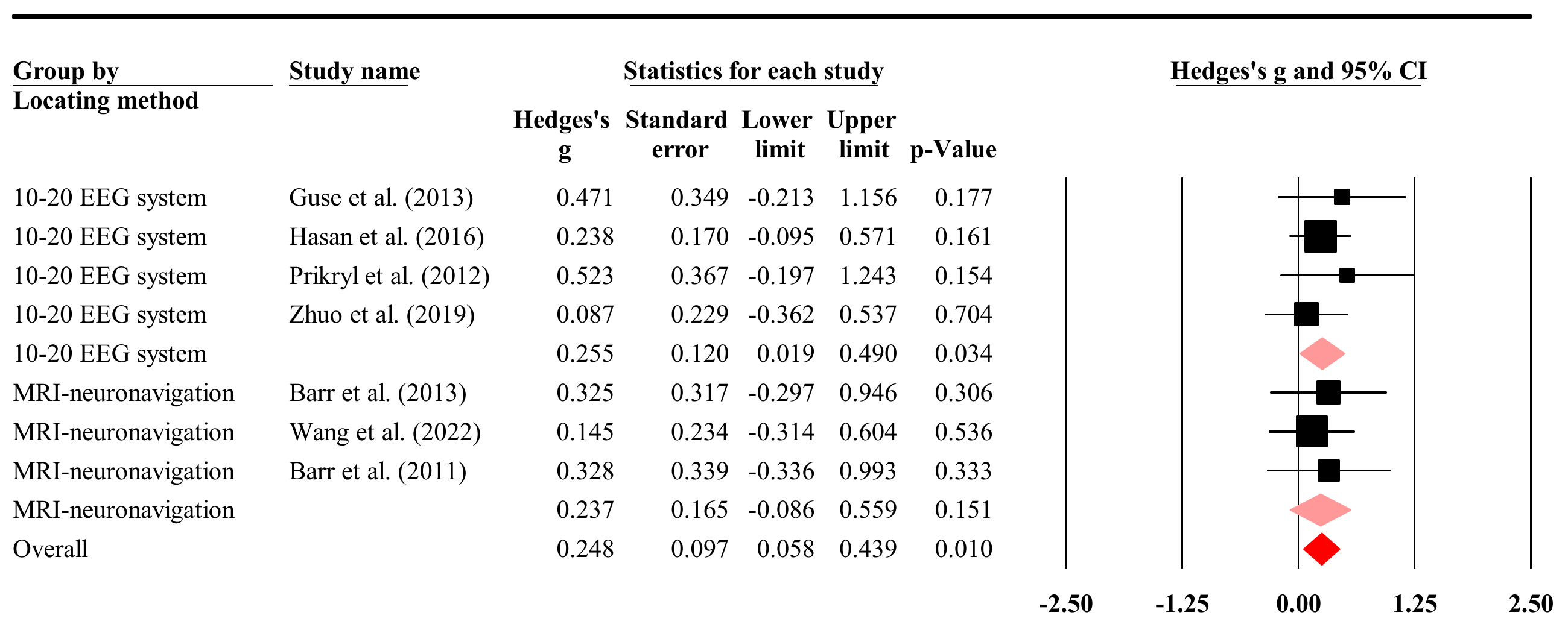


**Figure S6b.** Forest plot showing subgroup analyses for placebo effects grouped by methods of target localization in working memory.


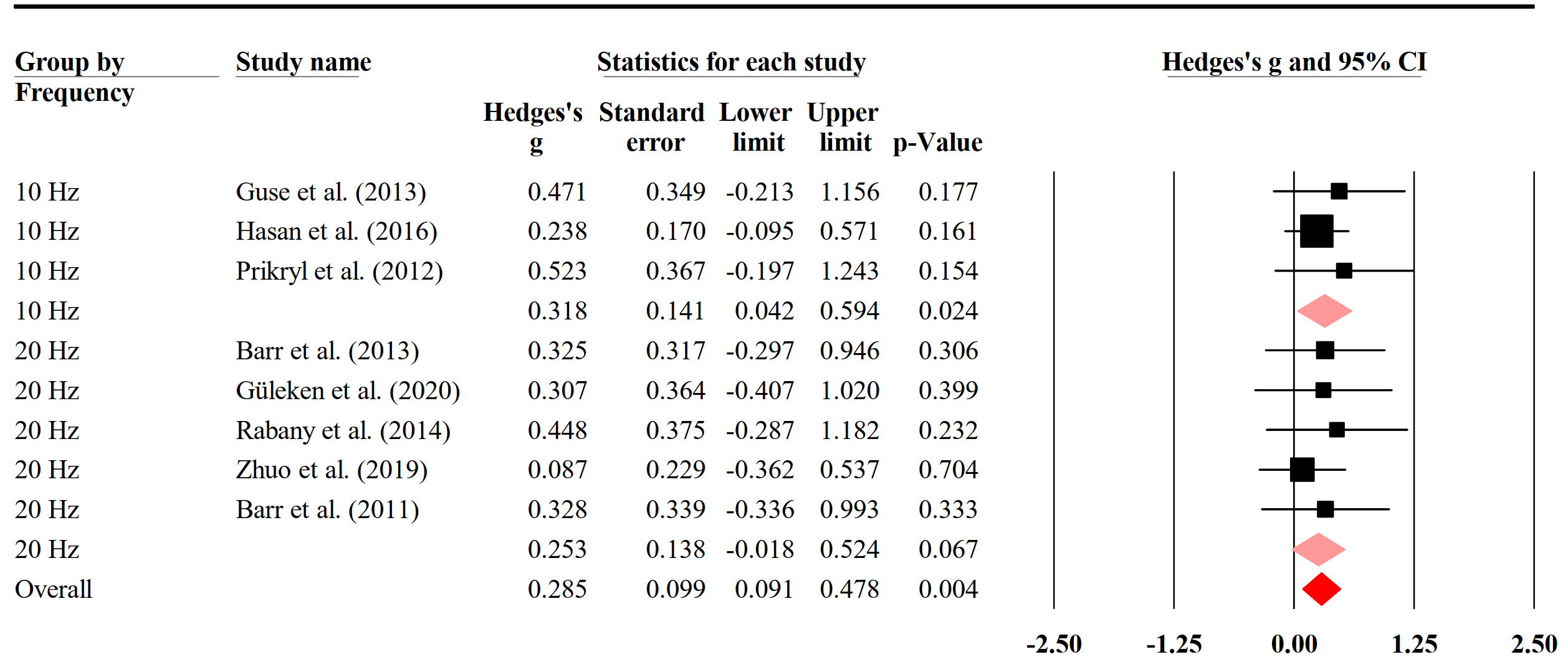


**Figure S6c.** Forest plot showing subgroup analyses for placebo effects grouped by frequency in working memory.


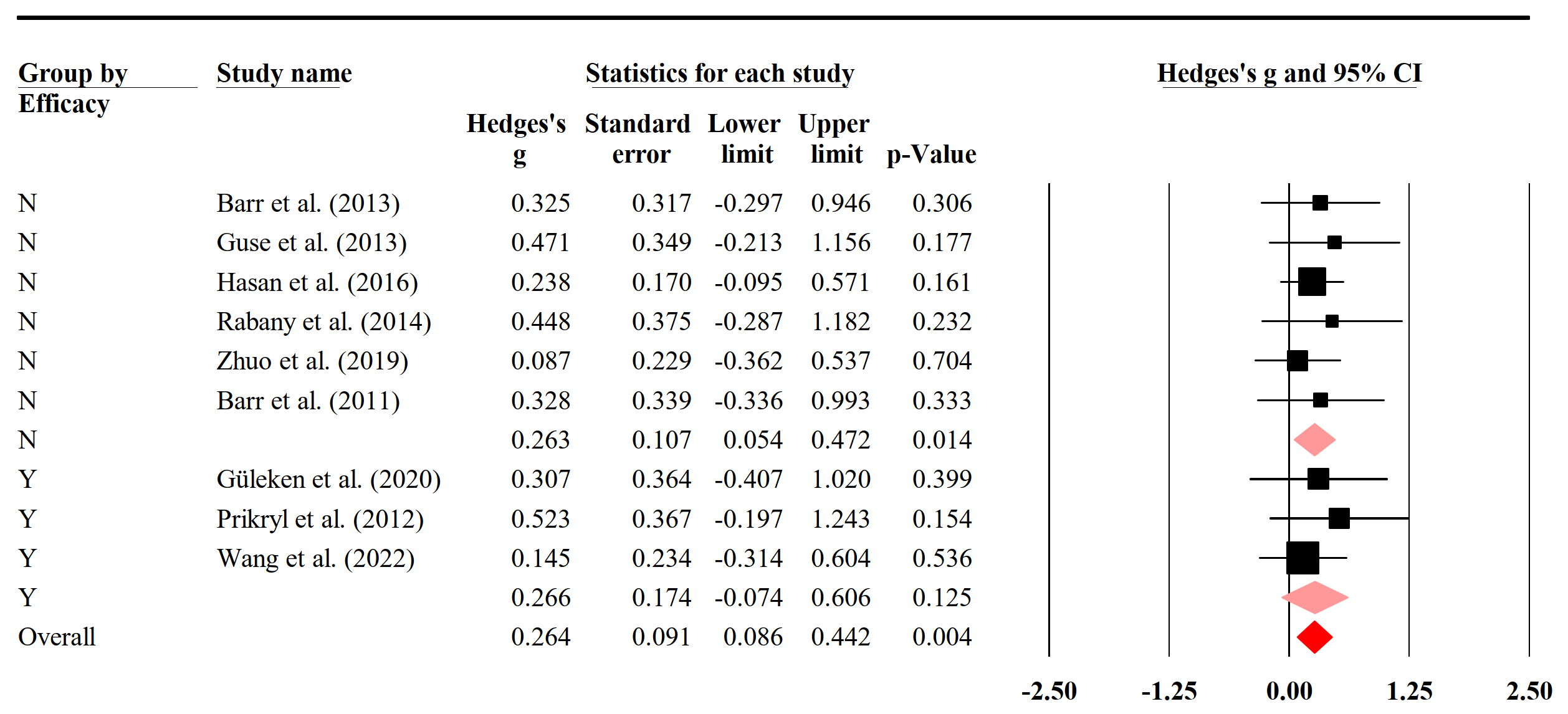


**Figure S6d.** Forest plot showing subgroup analyses for placebo effects grouped by efficacy of active rTMS over sham rTMS in working memory.

**Attention**


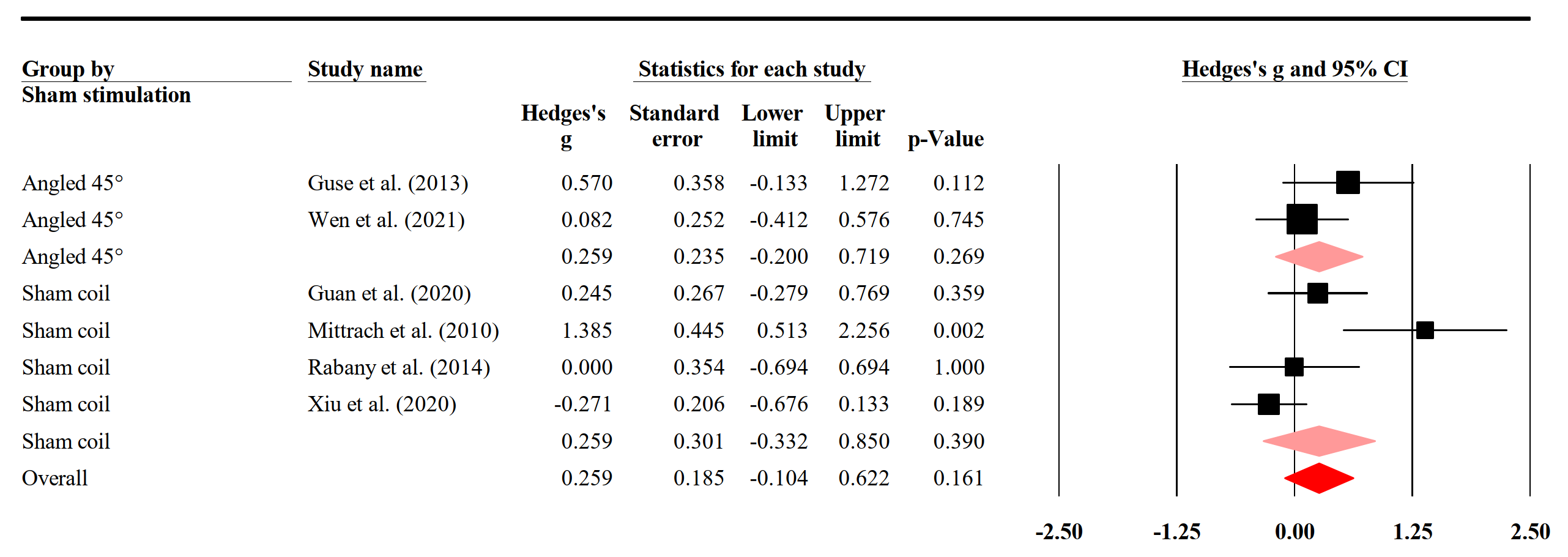


**Figure S7a.** Forest plot showing subgroup analyses for placebo effects grouped by sham conditions in attention.


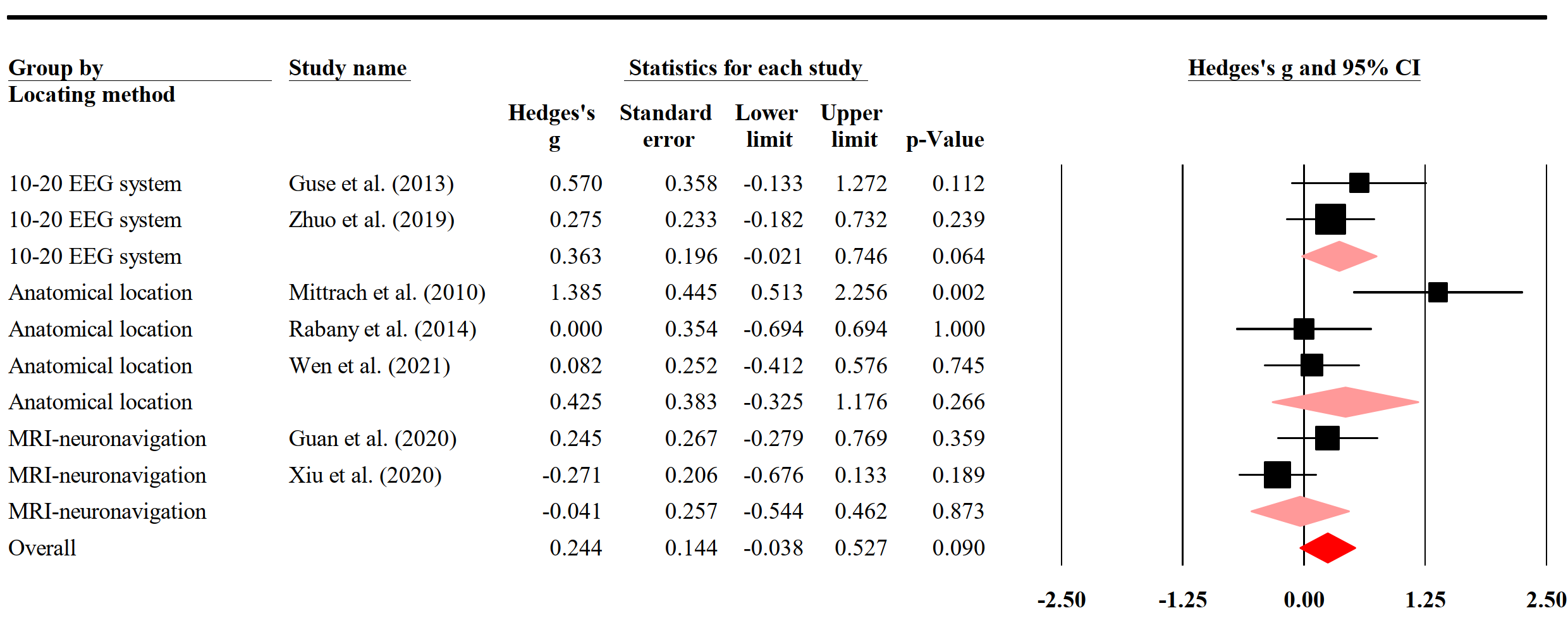


**Figure S7b.** Forest plot showing subgroup analyses for placebo effects grouped by methods of target localization in attention.


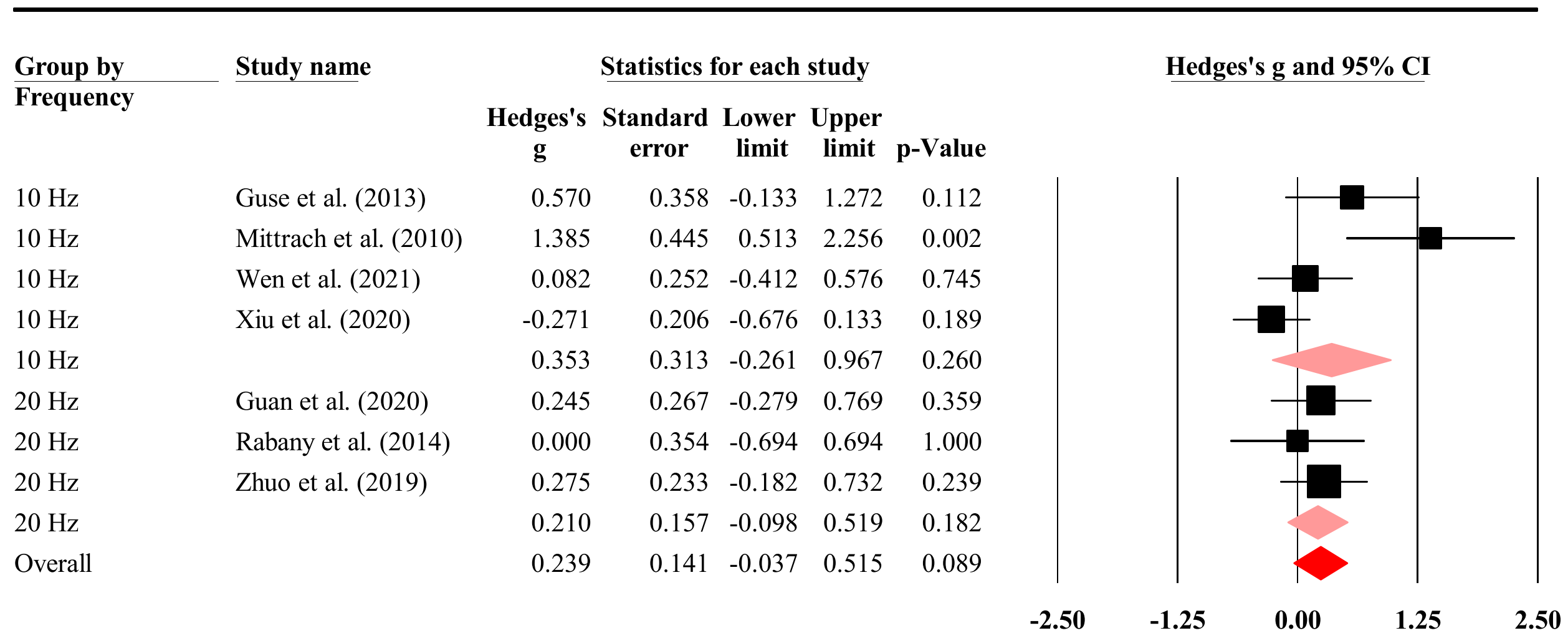


**Figure S7c.** Forest plot showing subgroup analyses for placebo effects grouped by frequency in attention.

**Processing speed**


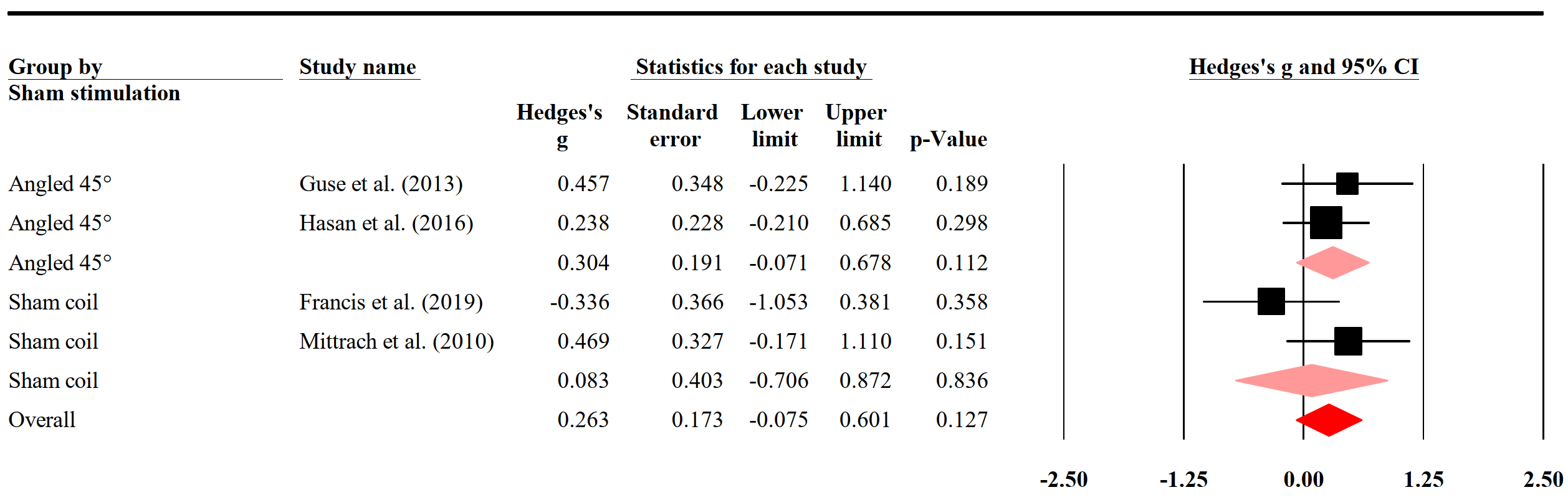


**Figure S8a.** Forest plot showing subgroup analyses for placebo effects grouped by sham

conditions in processing speed.


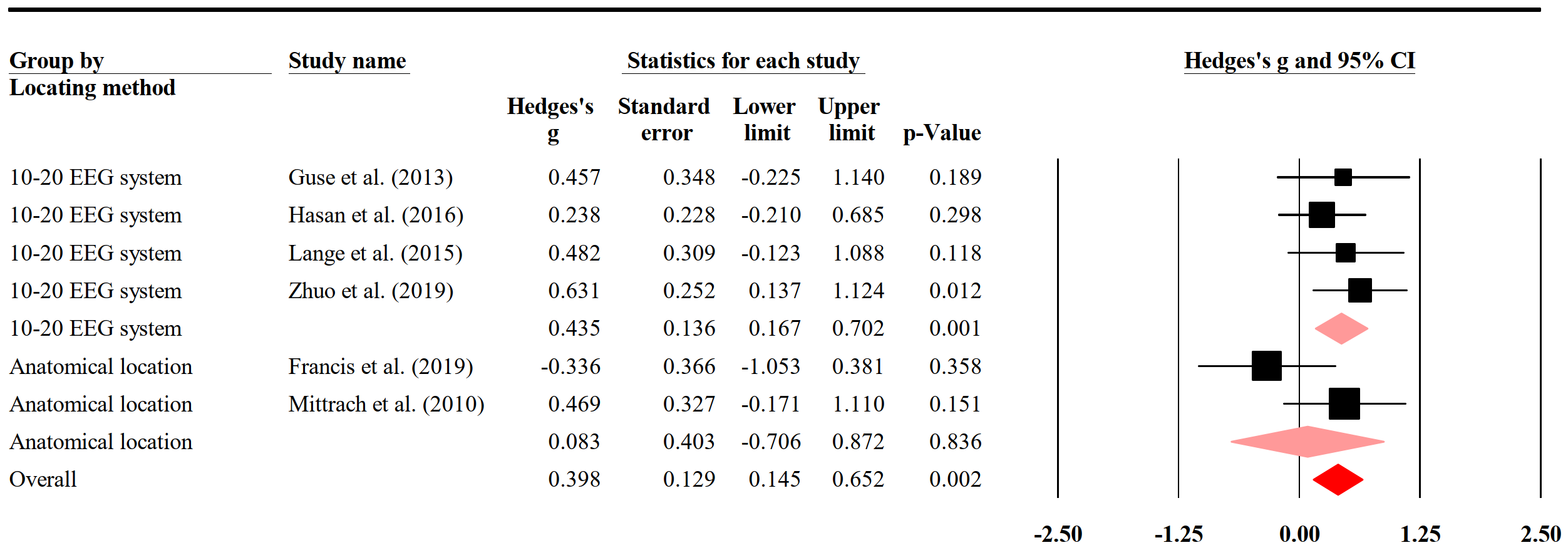


**Figure S8b.** Forest plot showing subgroup analyses for placebo effects grouped by methods of target localization in processing speed.


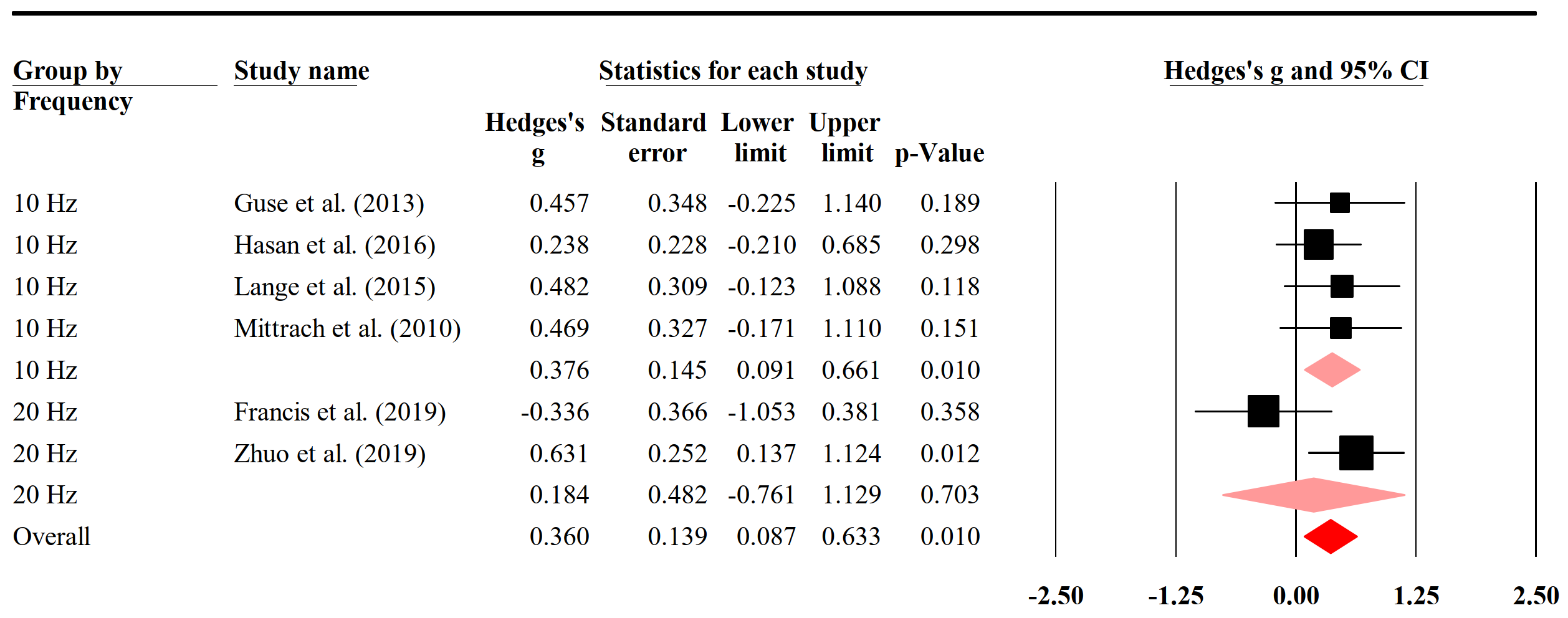


**Figure S8c.** Forest plot showing subgroup analyses for placebo effects grouped by frequency in processing speed.

**Full list of included studies**

1. Barr MS, Farzan F, Arenovich T, Chen R, Fitzgerald PB, Daskalakis ZJ. The effect of repetitive transcranial magnetic stimulation on gamma oscillatory activity in schizophrenia. *PLoS One* (2011) 6:e22627. doi: 10.1371/journal.pone.0022627

2. Barr MS, Farzan F, Tran LC, Fitzgerald PB, Daskalakis ZJ. A randomized controlled trial of sequentially bilateral prefrontal cortex repetitive transcranial magnetic stimulation in the treatment of negative symptoms in schizophrenia. *Brain Stimul* (2012) 5:337–346. doi: 10.1016/j.brs.2011.06.003

3. Barr MS, Farzan F, Rajji TK, Voineskos AN, Blumberger DM, Arenovich T, Fitzgerald PB, Daskalakis ZJ. Can repetitive magnetic stimulation improve cognition in schizophrenia? Pilot data from a randomized controlled trial. *Biol Psychiatry* (2013) 73:510–517. doi: 10.1016/j.biopsych.2012.08.020

4. Basavaraju R, Ithal D, Thanki MV, Ramalingaiah AH, Thirthalli J, Reddy RP, Brady RO, Halko MA, Bolo NR, Keshavan MS, et al. Intermittent theta burst stimulation of cerebellar vermis enhances fronto-cerebellar resting state functional connectivity in schizophrenia with predominant negative symptoms: A randomized controlled trial. *Schizophr Res* (2021) 238:108–120. doi: 10.1016/j.schres.2021.10.005

5. Bation R, Magnin C, Poulet E, Mondino M, Brunelin J. Intermittent theta burst stimulation for negative symptoms of schizophrenia-A double-blind, sham-controlled pilot study. *NPJ Schizophr* (2021) 7:10. doi: 10.1038/s41537-021-00138-3

6. Bodén R, Bengtsson J, Thörnblom E, Struckmann W, Persson J. Dorsomedial prefrontal theta burst stimulation to treat anhedonia, avolition, and blunted affect in schizophrenia or depression - a randomized controlled trial. *J Affect Disord* (2021) 290:308–315. doi: 10.1016/j.jad.2021.04.053

7. Chauhan P, Garg S, Tikka SK, Khattri S. Efficacy of Intensive Cerebellar Intermittent Theta Burst Stimulation (iCiTBS) in Treatment-Resistant Schizophrenia: a Randomized Placebo-Controlled Study. *Cerebellum* (2021) 20:116–123. doi: 10.1007/s12311-020-01193-9

8. Du X-D, Li Z, Yuan N, Yin M, Zhao X-L, Lv X-L, Zou S-Y, Zhang J, Zhang G-Y, Li C-W, et al. Delayed improvements in visual memory task performance among chronic schizophrenia patients after high-frequency repetitive transcranial magnetic stimulation. *World J Psychiatry* (2022) 12:1169–1182. doi: 10.5498/wjp.v12.i9.1169

9. Fitzgerald PB, Herring S, Hoy K, McQueen S, Segrave R, Kulkarni J, Daskalakis ZJ. A study of the effectiveness of bilateral transcranial magnetic stimulation in the treatment of the negative symptoms of schizophrenia. *Brain Stimul* (2008) 1:27–32. doi: 10.1016/j.brs.2007.08.001

10. Francis MM, Hummer TA, Vohs JL, Yung MG, Visco AC, Mehdiyoun NF, Kulig TC, Um M, Yang Z, Motamed M, et al. Cognitive effects of bilateral high frequency repetitive transcranial magnetic stimulation in early phase psychosis: a pilot study. *Brain Imaging Behav* (2019) 13:852–861. doi: 10.1007/s11682-018-9902-4

11. Garg S, Sinha VK, Tikka SK, Mishra P, Goyal N. The efficacy of cerebellar vermal deep high frequency (theta range) repetitive transcranial magnetic stimulation (rTMS) in schizophrenia: A randomized rater blind-sham controlled study. *Psychiatry Res* (2016) 243:413–420. doi: 10.1016/j.psychres.2016.07.023

12. Guan HY, Zhao JM, Wang KQ, Su XR, Pan YF, Guo JM, Jiang L, Wang YH, Liu HY, Sun SG, et al. High-frequency neuronavigated rTMS effect on clinical symptoms and cognitive dysfunction: a pilot double-blind, randomized controlled study in Veterans with schizophrenia. *Transl Psychiatry* (2020) 10:79. doi: 10.1038/s41398-020-0745-6

13. Güleken MD, Akbaş T, Erden SÇ, Akansel V, Al ZC, Özer ÖA. The effect of bilateral high frequency repetitive transcranial magnetic stimulation on cognitive functions in schizophrenia. *Schizophr Res Cogn* (2020) 22:100183. doi: 10.1016/j.scog.2020.100183

14. Guse B, Falkai P, Gruber O, Whalley H, Gibson L, Hasan A, Obst K, Dechent P, McIntosh A, Suchan B, et al. The effect of long-term high frequency repetitive transcranial magnetic stimulation on working memory in schizophrenia and healthy controls--a randomized placebo-controlled, double-blind fMRI study. *Behav Brain Res* (2013) 237:300–307. doi: 10.1016/j.bbr.2012.09.034

15. Hasan A, Guse B, Cordes J, Wölwer W, Winterer G, Gaebel W, Langguth B, Landgrebe M, Eichhammer P, Frank E, et al. Cognitive Effects of High-Frequency rTMS in Schizophrenia Patients With Predominant Negative Symptoms: Results From a Multicenter Randomized Sham-Controlled Trial. *Schizophr Bull* (2016) 42:608–618. doi: 10.1093/schbul/sbv142

16. Holi MM, Eronen M, Toivonen K, Toivonen P, Marttunen M, Naukkarinen H. Left prefrontal repetitive transcranial magnetic stimulation in schizophrenia. *Schizophr Bull* (2004) 30:429–434. doi: 10.1093/oxfordjournals.schbul.a007089

17. Huang W, Shen F, Zhang J, Xing B. Effect of Repetitive Transcranial Magnetic Stimulation on Cigarette Smoking in Patients with Schizophrenia. *Shanghai Arch Psychiatry* (2016) 28:309–317. doi: 10.11919/j.issn.1002-0829.216044

18. Jin Y, Tong J, Huang Y, Shi D, Zhu N, Zhu M, Liu M, Liu H, Sun X. Effectiveness of accelerated intermittent theta burst stimulation for social cognition and negative symptoms among individuals with schizophrenia: A randomized controlled trial. *Psychiatry Res* (2023) 320:115033. doi: 10.1016/j.psychres.2022.115033

19. Klein E, Kolsky Y, Puyerovsky M, Koren D, Chistyakov A, Feinsod M. Right prefrontal slow repetitive transcranial magnetic stimulation in schizophrenia: a double-blind sham-controlled pilot study. *Biol Psychiatry* (1999) 46:1451–1454. doi: 10.1016/s0006-3223(99)00182-1

20. Kumar N, Vishnubhatla S, Wadhawan AN, Minhas S, Gupta P. A randomized, double blind, sham-controlled trial of repetitive transcranial magnetic stimulation (rTMS) in the treatment of negative symptoms in schizophrenia. *Brain Stimul* (2020) 13:840–849. doi: 10.1016/j.brs.2020.02.016

21. Dlabac-de Lange JJ, Bais L, van Es FD, Visser BGJ, Reinink E, Bakker B, van den Heuvel ER, Aleman A, Knegtering H. Efficacy of bilateral repetitive transcranial magnetic stimulation for negative symptoms of schizophrenia: results of a multicenter double-blind randomized controlled trial. *Psychol Med* (2015) 45:1263–1275. doi: 10.1017/s0033291714002360

22. Li Z, Yin M, Lyu X-L, Zhang L-L, Du X-D, Hung GC-L. Delayed effect of repetitive transcranial magnetic stimulation (rTMS) on negative symptoms of schizophrenia: Findings from a randomized controlled trial. *Psychiatry Res* (2016) 240:333–335. doi: 10.1016/j.psychres.2016.04.046

23. McIntosh AM, Semple D, Tasker K, Harrison LK, Owens DGC, Johnstone EC, Ebmeier KP. Transcranial magnetic stimulation for auditory hallucinations in schizophrenia. *Psychiatry Research* (2004) 127:9–17. doi: 10.1016/j.psychres.2004.03.005

24. Mittrach M, Thünker J, Winterer G, Agelink MW, Regenbrecht G, Arends M, Mobascher A, Kim S-J, Wölwer W, Brinkmeyer J, et al. The tolerability of rTMS treatment in schizophrenia with respect to cognitive function. *Pharmacopsychiatry* (2010) 43:110–117. doi: 10.1055/s-0029-1242824

25. Mogg A, Purvis R, Eranti S, Contell F, Taylor JP, Nicholson T, Brown RG, McLoughlin DM. Repetitive transcranial magnetic stimulation for negative symptoms of schizophrenia: a randomized controlled pilot study. *Schizophr Res* (2007) 93:221–228. doi: 10.1016/j.schres.2007.03.016

26. Pan Z, Xiong D, Xiao H, Li J, Huang Y, Zhou J, Chen J, Li X, Ning Y, Wu F, et al. The Effects of Repetitive Transcranial Magnetic Stimulation in Patients with Chronic Schizophrenia: Insights from EEG Microstates. *Psychiatry Res* (2021) 299:113866. doi: 10.1016/j.psychres.2021.113866

27. Prikryl R, Kasparek T, Skotakova S, Ustohal L, Kucerova H, Ceskova E. Treatment of negative symptoms of schizophrenia using repetitive transcranial magnetic stimulation in a double-blind, randomized controlled study. *Schizophr Res* (2007) 95:151–157. doi: 10.1016/j.schres.2007.06.019

28. Prikryl R, Mikl M, Prikrylova Kucerová H, Ustohal L, Kasparek T, Marecek R, Vrzalova M, Ceskova E, Vanicek J. Does repetitive transcranial magnetic stimulation have a positive effect on working memory and neuronal activation in treatment of negative symptoms of schizophrenia? *Neuro Endocrinol Lett* (2012) 33:90–97.

29. Prikryl R, Ustohal L, Prikrylova Kucerova H, Kasparek T, Venclikova S, Vrzalova M, Ceskova E. A detailed analysis of the effect of repetitive transcranial magnetic stimulation on negative symptoms of schizophrenia: a double-blind trial. *Schizophr Res* (2013) 149:167–173. doi: 10.1016/j.schres.2013.06.015

30. Prikryl R, Ustohal L, Kucerova HP, Kasparek T, Jarkovsky J, Hublova V, Vrzalova M, Ceskova E. Repetitive transcranial magnetic stimulation reduces cigarette consumption in schizophrenia patients. *Prog Neuropsychopharmacol Biol Psychiatry* (2014) 49:30–35. doi: 10.1016/j.pnpbp.2013.10.019

31. Quan WX, Zhu XL, Qiao H, Zhang WF, Tan SP, Zhou DF, Wang XQ. The effects of high-frequency repetitive transcranial magnetic stimulation (rTMS) on negative symptoms of schizophrenia and the follow-up study. *Neurosci Lett* (2015) 584:197–201. doi: 10.1016/j.neulet.2014.10.029

32. Rabany L, Deutsch L, Levkovitz Y. Double-blind, randomized sham controlled study of deep-TMS add-on treatment for negative symptoms and cognitive deficits in schizophrenia. *J Psychopharmacol* (2014) 28:686–690. doi: 10.1177/0269881114533600

33. Saba G, Verdon CM, Kalalou K, Rocamora JF, Dumortier G, Benadhira R, Stamatiadis L, Vicaut E, Lipski H, Januel D. Transcranial magnetic stimulation in the treatment of schizophrenic symptoms: a double blind sham controlled study. *J Psychiatr Res* (2006) 40:147–152. doi: 10.1016/j.jpsychires.2005.02.008

34. Singh S, Kumar N, Verma R, Nehra A. The safety and efficacy of adjunctive 20-Hz repetitive transcranial magnetic stimulation for treatment of negative symptoms in patients with schizophrenia: A double-blinded, randomized, sham-controlled study. *Indian J Psychiatry* (2020) 62:21–29. doi: 10.4103/psychiatry.indianjpsychiatry_361_19

35. Su X, Zhao L, Shang Y, Chen Y, Liu X, Wang X, Xiu M, Yu H, Liu L. Repetitive transcranial magnetic stimulation for psychiatric symptoms in long-term hospitalized veterans with schizophrenia: A randomized double-blind controlled trial. *Front Psychiatry* (2022) 13:873057. doi: 10.3389/fpsyt.2022.873057

36. Tikka SK, Nizamie SH, Venkatesh Babu GM, Aggarwal N, Das AK, Goyal N. Safety and Efficacy of Adjunctive Θ Burst Repetitive Transcranial Magnetic Stimulation to Right Inferior Parietal Lobule in Schizophrenia Patients With First-Rank Symptoms: A Pilot, Exploratory Study. *J ECT* (2017) 33:43–51. doi: 10.1097/yct.0000000000000343

37. Wang L, Li Q, Wu Y, Ji G-J, Wu X, Xiao G, Qiu B, Hu P, Chen X, He K, et al. Intermittent theta burst stimulation improved visual-spatial working memory in treatment-resistant schizophrenia: A pilot study. *J Psychiatr Res* (2022) 149:44–53. doi: 10.1016/j.jpsychires.2022.02.019

38. Wen N, Chen L, Miao X, Zhang M, Zhang Y, Liu J, Xu Y, Tong S, Tang W, Wang M, et al. Effects of High-Frequency rTMS on Negative Symptoms and Cognitive Function in Hospitalized Patients With Chronic Schizophrenia: A Double-Blind, Sham-Controlled Pilot Trial. *Front Psychiatry* (2021) 12:736094. doi: 10.3389/fpsyt.2021.736094

39. Wobrock T, Guse B, Cordes J, Wölwer W, Winterer G, Gaebel W, Langguth B, Landgrebe M, Eichhammer P, Frank E, et al. Left prefrontal high-frequency repetitive transcranial magnetic stimulation for the treatment of schizophrenia with predominant negative symptoms: a sham-controlled, randomized multicenter trial. *Biol Psychiatry* (2015) 77:979–988. doi: 10.1016/j.biopsych.2014.10.009

40. Wölwer W, Lowe A, Brinkmeyer J, Streit M, Habakuck M, Agelink MW, Mobascher A, Gaebel W, Cordes J. Repetitive transcranial magnetic stimulation (rTMS) improves facial affect recognition in schizophrenia. *Brain Stimul* (2014) 7:559–563. doi: 10.1016/j.brs.2014.04.011

41. Xiu MH, Guan HY, Zhao JM, Wang KQ, Pan YF, Su XR, Wang YH, Guo JM, Jiang L, Liu HY, et al. Cognitive Enhancing Effect of High-Frequency Neuronavigated rTMS in Chronic Schizophrenia Patients With Predominant Negative Symptoms: A Double-Blind Controlled 32-Week Follow-up Study. *Schizophr Bull* (2020) 46:1219–1230. doi: 10.1093/schbul/sbaa035

42. Zhao S, Kong J, Li S, Tong Z, Yang C, Zhong H. Randomized controlled trial of four protocols of repetitive transcranial magnetic stimulation for treating the negative symptoms of schizophrenia. *Shanghai Arch Psychiatry* (2014) 26:15–21. doi: 10.3969/j.issn.1002-0829.2014.01.003

43. Zhu L, Zhang W, Zhu Y, Mu X, Zhang Q, Wang Y, Cai J, Xie B. Cerebellar theta burst stimulation for the treatment of negative symptoms of schizophrenia: A multicenter, double-blind, randomized controlled trial. *Psychiatry Res* (2021) 305:114204. doi: 10.1016/j.psychres.2021.114204

44. Zhuo K, Tang Y, Song Z, Wang Y, Wang J, Qian Z, Li H, Xiang Q, Chen T, Yang Z, et al. Repetitive transcranial magnetic stimulation as an adjunctive treatment for negative symptoms and cognitive impairment in patients with schizophrenia: a randomized, double-blind, sham-controlled trial. *Neuropsychiatr Dis Treat* (2019) 15:1141–1150. doi: 10.2147/ndt.s196086
